# Clinical relevance of loss-of-function mutations of *NEMO*/*IKBKG*

**DOI:** 10.1016/j.gendis.2025.101531

**Published:** 2025-01-12

**Authors:** Jin Wang, Kexin Shen, Hongxia Lou, Lina Zhou, Yunfei An, Xiaodong Zhao, Yuan Ding

**Affiliations:** a Growth, Development and Mental Health Center of Children and Adolescents, Children’s Hospital of Chongqing Medical University, Chongqing Key Laboratory of Child Neurodevelopment and Cognitive Disorders, National Clinical Research Center for Child Health and Disorders, Ministry of Education Key Laboratory of Child Development and Disorders, Chongqing 401146, China; b Chongqing Key Laboratory of Child Infection and Immunity, Children's Hospital of Chongqing Medical University, Chongqing 400014, China

**Keywords:** Anhidrotic ectodermal dysplasia with immunodeficiency (EDA-ID), Immunodeficiency (ID), Incontinentia pigmenti (IP), Inhibitor of nuclear factor kappa B kinase regulatory subunit gamma (IKBKG), NEMO deleted exon 5 autoinflammatory syndrome (NDAS), Nuclear factor-κB (NF-κB) essential modulator (NEMO)

## Abstract

Dysfunctional inhibitor of nuclear factor-κB (NF-κB) kinase regulatory subunit gamma (*IKBKG*) is known to trigger incontinentia pigmenti (IP), anhidrotic ectodermal dysplasia with immunodeficiency (EDA-ID), immunodeficiency (ID), and *IKBKG* deleted exon 5 autoinflammatory syndrome (NDAS). The correlation between genotype and phenotype remains elusive because of the considerable variability in *IKBKG* genes. This study aimed to systematically describe *IKBKG* gene mutations and clinical characteristics. Cases with *IKBKG* mutations and thorough clinical features were gathered using PubMed, Web of Science, EMBASE, Scopus, and Cochrane databases, with a publication deadline of February 12, 2023. The Newcastle-Ottawa scale and its modified version were used to assess the quality of each study. Gene mutations and clinical manifestation data were analyzed and reviewed. 144 publications with 564 patients were included in the analysis. IP, EDA-ID, ID, and NDAS accounted for 78.0%, 15.8%, 5.0%, and 1.2% of *IKBKG* mutations, respectively. Skin abnormalities (89.5%), dental abnormalities (68.5%), infection (100%), and non-infectious inflammation (100%) were the most common manifestations of IP, EDA-ID, ID, and NDAS, respectively. Mutations related to EDA-ID and ID are concentrated in the zinc finger region and characterized by the most severe clinical symptoms. E390RfsX5 can cause IP, EDA-ID, and ID. c.1182_1183delTT and H413R caused the most clinical manifestations. *Mycobacterium* (22.7%) and *Streptococcus* (17.5%) were the most common pathogens. Almost all cases of hyper-IgM occurred in patients with EDA-ID. Different structural domains correspond to symptoms with varying degrees of severity. Certain mutations may correspond to unique manifestations, providing insight into disease progression.

## Introduction

The nuclear factor-κB (NF-κB) pathway, a signaling pathway of notable conservation in biological evolution, is instrumental in various cellular processes, including survival, proliferation, activation, differentiation, aging, and death.^1^ The inhibitor of NF-κB kinase regulatory subunit gamma (*IKBKG*, Online Mendelian Inheritance in Man (OMIM) 300248), also known as the NF-κB essential modulator (*NEMO)*, serves as a pivotal regulatory subunit in the NF-κB signaling pathway, forming the IKK complex with IKKα and IKKβ and participating in the activation of the NF-κB signaling pathway.^2^ Mutations in *IKBKG* can cause varying degrees of inactivation of the NF-κB signaling pathway, leading to different clinical manifestations; when the NF-κB pathway undergoes *IKBKG* amorphic mutations, it can cause incontinentia pigmenti (IP, OMIM 308300), which is an X-linked dominant genetic disease mainly occurring in female but lethal in male.^3^ Some males survive because of the presence of an additional X chromosome, known as Klinefelter syndrome, or the existence of somatic mosaicism or hypomorphic mutations.^4^ Because the physiological process of ectodermal development relies on the regulatory factor NEMO in the NF-κB pathway,^5^ mutations in *IKBKG* can result in significant ectodermal dysplasia manifestations. These include pigmentation abnormalities of the skin, which are the primary features of IP. Additionally, both IP and anhidrotic ectodermal dysplasia with immunodeficiency share features such as morphological abnormalities of teeth, alopecia, thin hair, and nail dystrophy. Furthermore, anhidrotic ectodermal dysplasia with immunodeficiency commonly presents with sweat gland deficiency. Characteristic gene mutations of IP are represented by exon 4–10 rearrangements.^6^ In addition, NF-κB pathway can cause IP or anhidrotic ectodermal dysplasia with immunodeficiency (EDA-ID, OMIM 300291) when undergoes hypomorphic mutations in *IKBKG*; EDA-ID is an X-linked recessive inheritance often occurring in male,^7^ and its clinical manifestations encompass not only the aforementioned ectodermal dysplasia but also include immunodeficiency. Because of the important role of NF-κB in osteoclasts, specific *IKBKG* mutation sites can also lead to a special type of EDA-ID, namely osteopetrosis, lymphedema, EDA, and immunodeficiency (OL-EDA-ID, OMIM 300301),^8^ this is the most severe clinical phenotype caused by hypomorphic mutations in *IKBKG*. Current understanding suggests that osteopetrosis caused by *IKBKG* mutations results from impaired activation of the IKK complex in osteoclasts, disrupting their differentiation and function, and causing an imbalance between bone formation and resorption.^9^^,^^10^ However, the contribution of concurrent RANK signaling impairment or abnormal activation of other signaling pathways to osteopetrosis cannot be excluded. Additionally, lymphoedema in OL-EDA-ID may result from abnormalities in the NEMO-dependent, VEGF-3-mediated NF-κB signaling pathway.^8^ In addition, dysregulation of the NF-κB pathway can cause disorders in Toll-like receptor, B cell receptor, and T cell receptor signal transduction, leading to immunodeficiency (ID, OMIM 300636) without ectodermal dysplasia, which is mainly manifested by recurrent infections.^11^ *NEMO* deleted exon 5 autoinflammatory syndrome (NDAS), also called X-linked systemic autoinflammatory disease (SAIDX, OMIM 301081), a new disease classification discovered in recent years,^12^ which mainly manifested by uveitis, lymphohistiocytic panniculitis, and hepatitis.

The phenotypes of *IKBKG* mutations are highly diverse. However, to date, comprehensive reviews have yet to address *IKBKG* mutations. This article aimed to systematically review and categorize cases associated with *IKBKG* mutations up to February 12, 2023, and delineate the clinical characteristics pertinent to these mutations.

## Methods

### Protocol and registration

This systematic review was conducted in accordance with the Preferred Reporting Items for Systematic Reviews and Meta-Analyses (PRISMA) guideline,^13^ and the protocol was registered in the PROSPERO database (authorized ID number: CRD42023405264).

### Search strategy

The following keywords were searched using Medical Subject Headings (MeSH) in PubMed: “NEMO”, “IKBKG”, “IP”, “EDA-ID”, “IMD33″, and “SAIDX”, and then formed into an appropriate search strategy using logical words “and” and “or” to form logical connections, and executed in PubMed, Web of Science, EMBASE, Scopus, and Cochrane with a publication deadline of February 12, 2023, and the language of English. Additional relevant but excluded studies were manually searched using Google Scholar.

### Study selection and screening

Initially, EndNote was used to eliminate duplicate studies, and the remaining articles were assessed by reviewing their abstracts. Letters, editorials, commentaries, books, meeting abstracts, guidelines, basic medical science experiments, and studies unrelated to *IKBKG* mutations or those lacking detailed clinical or laboratory information were excluded. Only studies reporting clinical or molecular characteristics associated with *IKBKG* mutations were included. Following this, a comprehensive full-text review was conducted on all articles to exclude those that failed to meet the predefined criteria.

### Data extraction and quality assessment

All included studies used Excel to extract patient sex, country, age at onset, age at gene test, age at diagnosis, age at death, symptoms, family histories, mutation sites, and therapy. The data extraction procedure was performed independently by Jin Wang and Kexin Shen, with any discrepancies being resolved by consensus. The methodological quality of case reports or case series was evaluated according to the Newcastle-Ottawa scale (NOS) and its modified version.^14^ Additionally, the NOS was used to evaluate case–control and cohort studies' quality and risk of bias (Table S1).

## Results

### Search result and study selection

**Figure 1:**
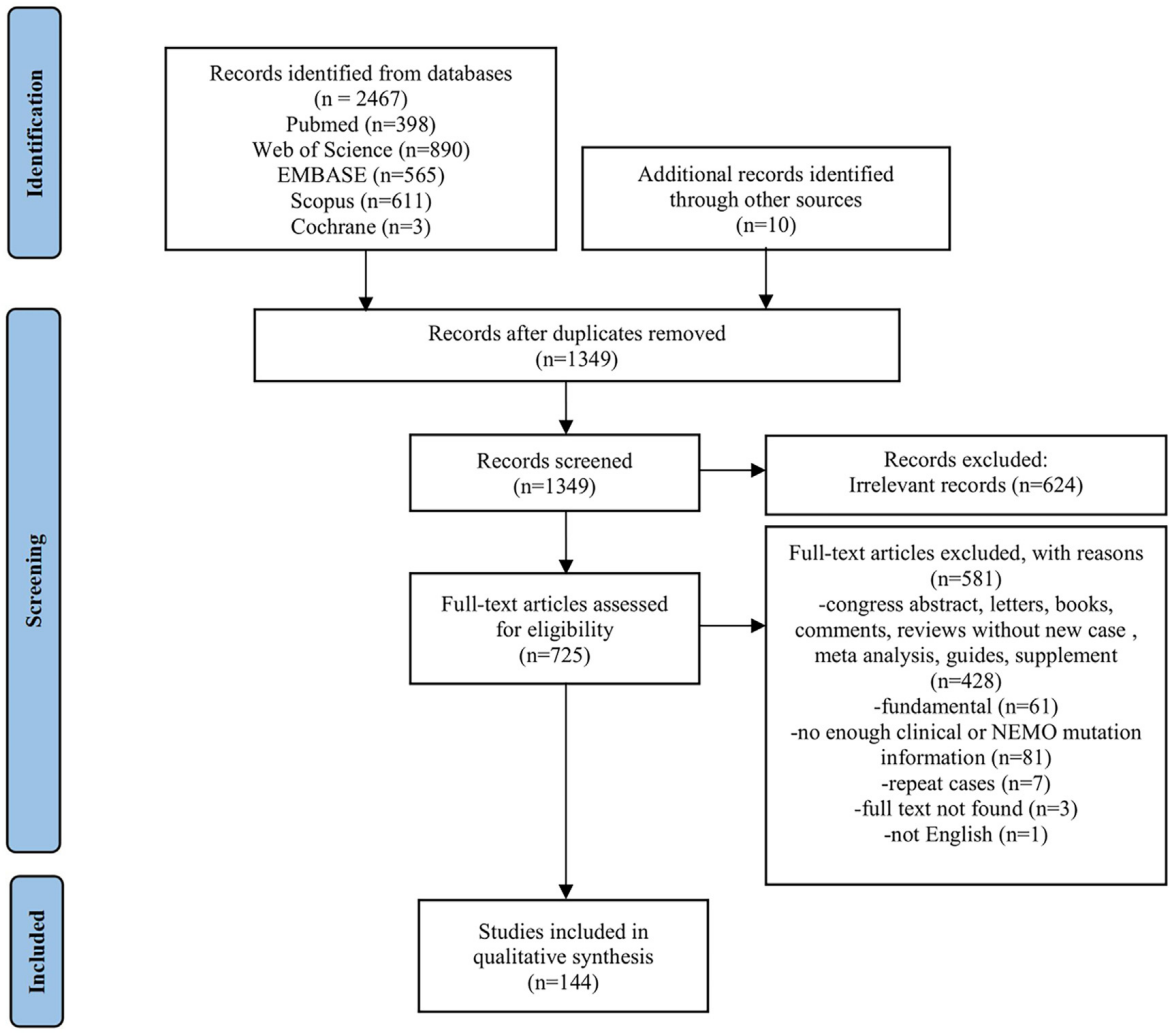
PRISMA flow diagram.

A total of 2477 studies were screened, of which 1128 repeated articles were excluded by EndNote and manual checks, and 624 irrelevant articles were excluded by reading the titles and abstracts. The remaining articles were retrieved by full-text reading; 428 conformed to the exclusion criteria for document type, 61 were fundamental articles, 81 lacked sufficient information on clinical or *IKBKG* mutation, seven were repeated cases, and the remaining four were unavailable in full-text and non-English. In total, 144 articles were included (Fig. 1).

### Study characteristics and patient demographics

The study included 144 articles covering 564 patients with *IKBKG* mutations, of whom the majority were patients with IP (440, 78.0%). Diseases caused by *IKBKG* mutations exhibit differences in sex composition. For instance, patients with IP are predominantly female (94.7%), whereas patients with EDA-ID (100%), ID (100%), and NDAS (100%) were male. The mutation was most common in France, the USA, Australia, and Japan. The age of onset varied among diseases. The disease developed after birth in 72.2% of patients with IP, while most patients with EDA-ID and all eight patients with ID and a clear onset time developed the disease after a month. In terms of disease diagnosis, 84.4% of patients with IP could be diagnosed during the neonatal period. Among the five patients with ID and a clear diagnosis time, three were diagnosed between one month and one year of age; more than half of patients with IP completed genetic testing at the age of one; most patients with EDA-ID died after reaching the age of one. The median age at death from EDA-ID was 3.5 years of age. In addition, for patients with IP and ID, family histories and sporadic cases accounted for approximately half of the cases. Among the patients with EDA-ID, those with a family history accounted for a larger proportion (71.2%), and all three patients with NDAS were sporadic cases (Table 1).

**Table 1:** Demographic data of patients with *NEMO* mutation.

		IP		EDA-ID		ID		NDAS	
No. of evaluated patients		n = 440		n = 89		n = 28		n = 7	
Sex, n (%) n; NK	M	22 (5.3)	*n* = 418 NK = 22	76 (98.7)	*n* = 77 NK = 12	27 (100)	*n* = 27 NK = 1	4 (100)	*n* = 4 NK = 3
	F	396 (94.7)		1 (1.3)		0 (0)		0 (0)	
Country, n (%) n; NK		France = 56 (18.1); Austria = 54 (17.5); Korea = 30 (9.7); China = 29 (9.4); Japan = 23 (7.4); Canada = 23 (7.4); Italy = 22 (7.1); Brazil = 15 (4.9); Greece = 12 (3.9); Serbia = 10 (3.2); Denmark = 8 (2.6); USA = 6 (1.9); German = 3 (0.9); Turkey = 3 (0.9); UK = 2 (0.6); Others = 13 (4.2);	n = 309 NK = 131	USA = 31 (40.8); Japan = 11 (14.5); France = 8 (10.5); German = 6 (7.9); UK = 5 (6.6); Italy = 4 (5.3); Turkey = 2 (2.6); Norway = 2 (2.6); Others = 7 (9.2);	*n* = 76 NK = 13	USA = 16 (59.3); UK = 5 (18.5); Japan = 2 (7.4); Turkey = 2 (7.4); German = 2 (7.4);	*n* = 27 NK = 1	USA = 7 (100);	*n* = 7 NK = 0
Age at onset, n (%) n; NK	<1Mo	57 (72.2)	*n* = 79 NK = 361	6 (23.1)	*n* = 26 NK = 63	0 (0)	*n* = 8 NK = 20	/	/
	1Mo-1Yr	17 (21.5)		10 (38.5)		4 (50)		/	
	≥1Yr	5 (6.3);		10 (38.5)		4 (50)		/	
Age at diagnosis, n (%) n; NK	<1Mo	27 (84.4)	*n* = 32 NK = 408	/	/	0 (0)	*n* = 5 NK = 23	/	/
	1Mo-1Yr	2 (6.3)		/		3 (60)		/	
	≥1Yr	3 (9.4)		/		2 (40)		/	
Age at gene test, n (%) n; NK	<1Mo	1 (3.6)	*n* = 28 NK = 412	/	/	/	/	/	/
	1Mo-1Yr	10 (35.7)		/		/		/	
	≥1Yr	17 (60.7)		/		/		/	
Age at death, n (%), n; NK	<1Mo	/	/	0 (0)	*n* = 20 NK = 69	/	/	/	/
	1Mo-1Yr	/		1 (7.7)		/		/	
	≥1Yr	/		19 (92.3)		/		/	
Family history, n (%), n; NK	Y	140 (50.2)	*n* = 279 NK = 161	37 (71.2)	*n* = 52 NK = 37	11 (50)	*n* = 22 NK = 6	0 (0)	*n* = 3 NK = 4
	S	139 (49.8)		15 (28.8)		11 (50)		3 (100)	

NK: not known, M:male, F: female, Y: yes, S: sporadic, Mo: month, Yr: year.

### Clinical characteristics of patients with *IKBKG* mutations

**Figure 2:**
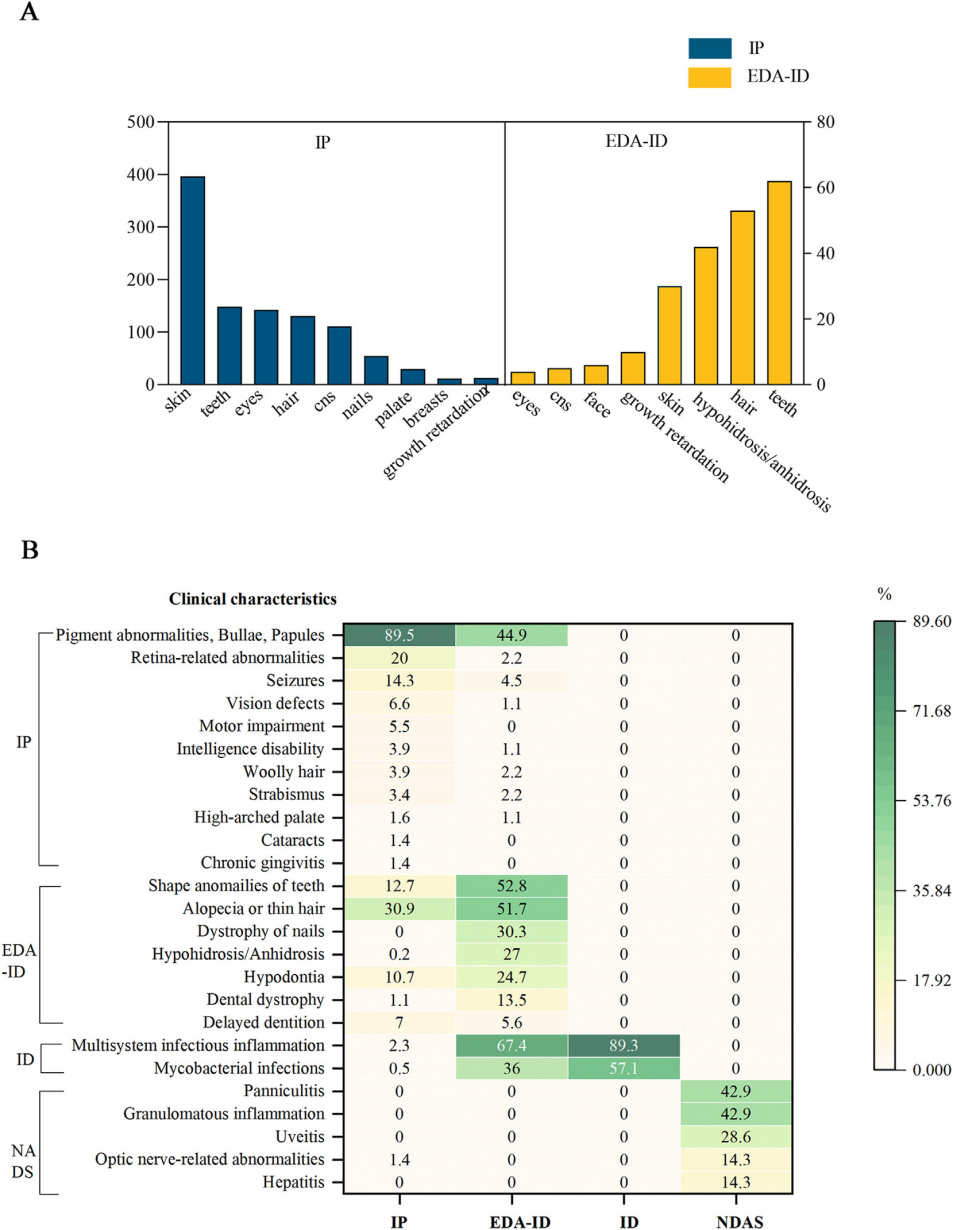
Clinical manifestations of *NEMO* mutations. **(A)** Comparison of ectodermal manifestations of IP and EDA-ID patients with *NEMO* mutations. **(B)** Heat map of detailed classification list of clinical findings for patients in IP, EDA-ID, ID, and NDAS with *NEMO* mutations. NEMO, nuclear factor-κB essential modulator; IP, incontinentia pigmenti; EDA-ID, anhidrotic ectodermal dysplasia with immunodeficiency; ID, immunodeficiency; NDAS, *IKBKG* deleted exon 5 autoinflammatory syndrome.

Most patients with IP (89.5%) presented with abnormal skin manifestations, notably hyperpigmentation or hypopigmentation. Additional prevalent symptoms included dental abnormalities (33.8%), such as abnormal tooth shape, missing teeth, and delayed dentition; ocular abnormalities (32.5%), including retinal-related abnormalities, vision defects, strabismus, cataracts, and optic nerve-related abnormalities; hair abnormalities (29.8%), such as alopecia, thin hair, and woolly hair; and central nervous system abnormalities (25.2%), such as seizures, motor impairment, intellectual disability, and developmental delay. Other less common symptoms include abnormalities in the nail, palate, and chest. Patients with EDA-ID typically exhibited dental (69.7%) and hair (59.6%) abnormalities, hypohidrosis or anhidrosis (47.2%), and skin (33.7%) abnormalities. Abnormalities of the central nervous system, eyes, and face were comparatively infrequent in these patients (Fig. 2).

**Figure 3:**
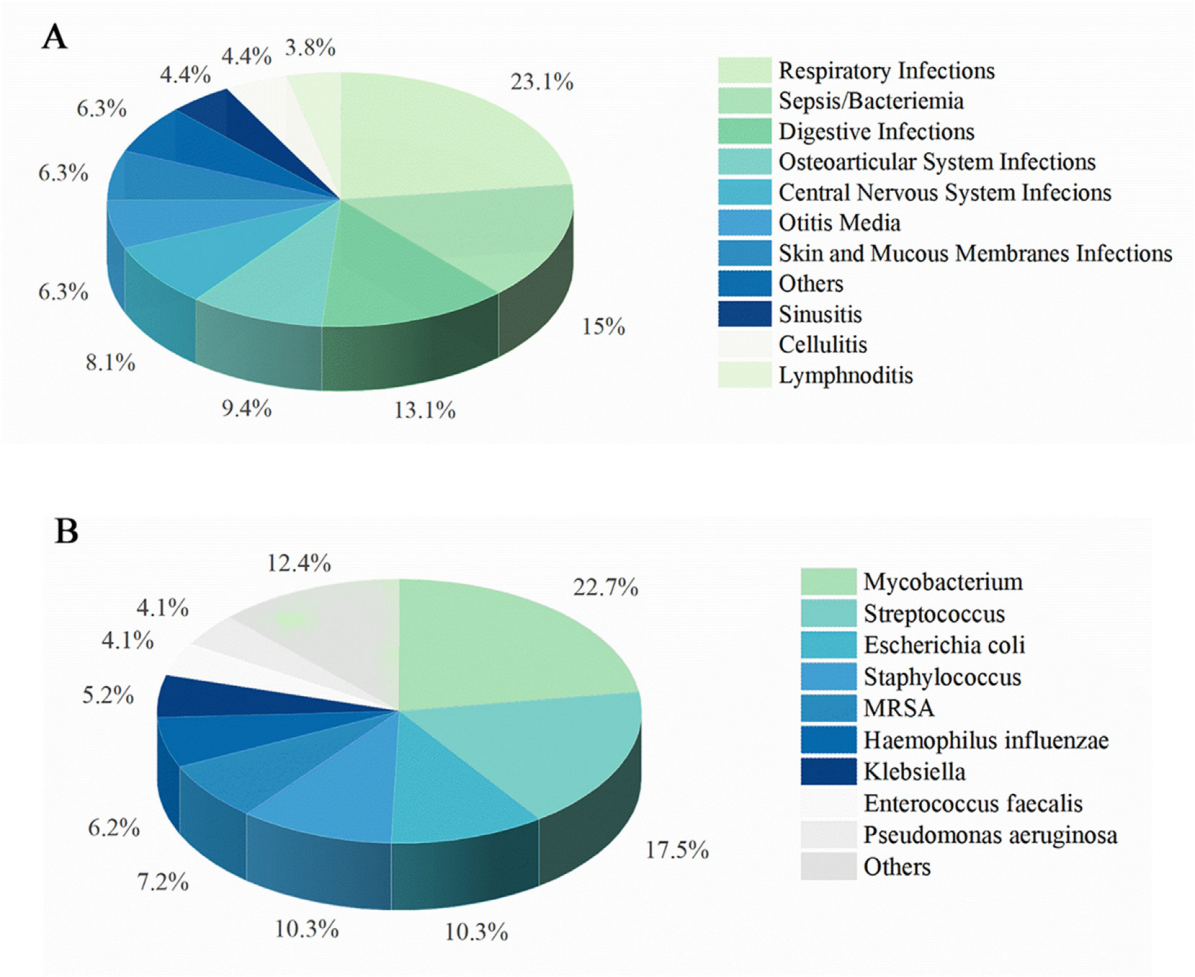
Infection in *NEMO* mutations. **(A)** Organ system inflammation caused by infection in patients with *NEMO* mutations. **(B)** Common bacterial pathogens in patients with *NEMO* mutations. NEMO, nuclear factor-κB essential modulator.

ID and NDAS represent infectious and non-infectious inflammation, respectively. Our analysis of the systems affected by inflammation due to *IKBKG* mutations revealed that the respiratory system was the most susceptible, followed by the hematological and digestive systems. It is crucial to note that minor clinical presentations, such as recurrent sinusitis and otitis media, may have been overlooked. This is particularly applicable to patients with ID lacking other characteristic manifestations, who are frequently misdiagnosed in the early stages of the disease (Fig. 3A). Almost a third of patients with *IKBKG* mutations had concomitant infections, with bacterial infections being the most common (79.7%), followed by viral (14.5%), fungal (5.1%), and parasitic (0.7%) infections. Among bacterial infections, the primary pathogens were *Mycobacteria*, *Streptococcus*, *Staphylococcus*, and *Escherichia coli*. Additionally, methicillin-resistant *Staphylococcus aureus* (MRSA), *Klebsiella*, *Enterococcus faecalis*, *Haemophilus influenzae*, and *Pseudomonas aeruginosa* accounted for a marked proportion of the cases (Fig. 3B).

**Figure 4:**
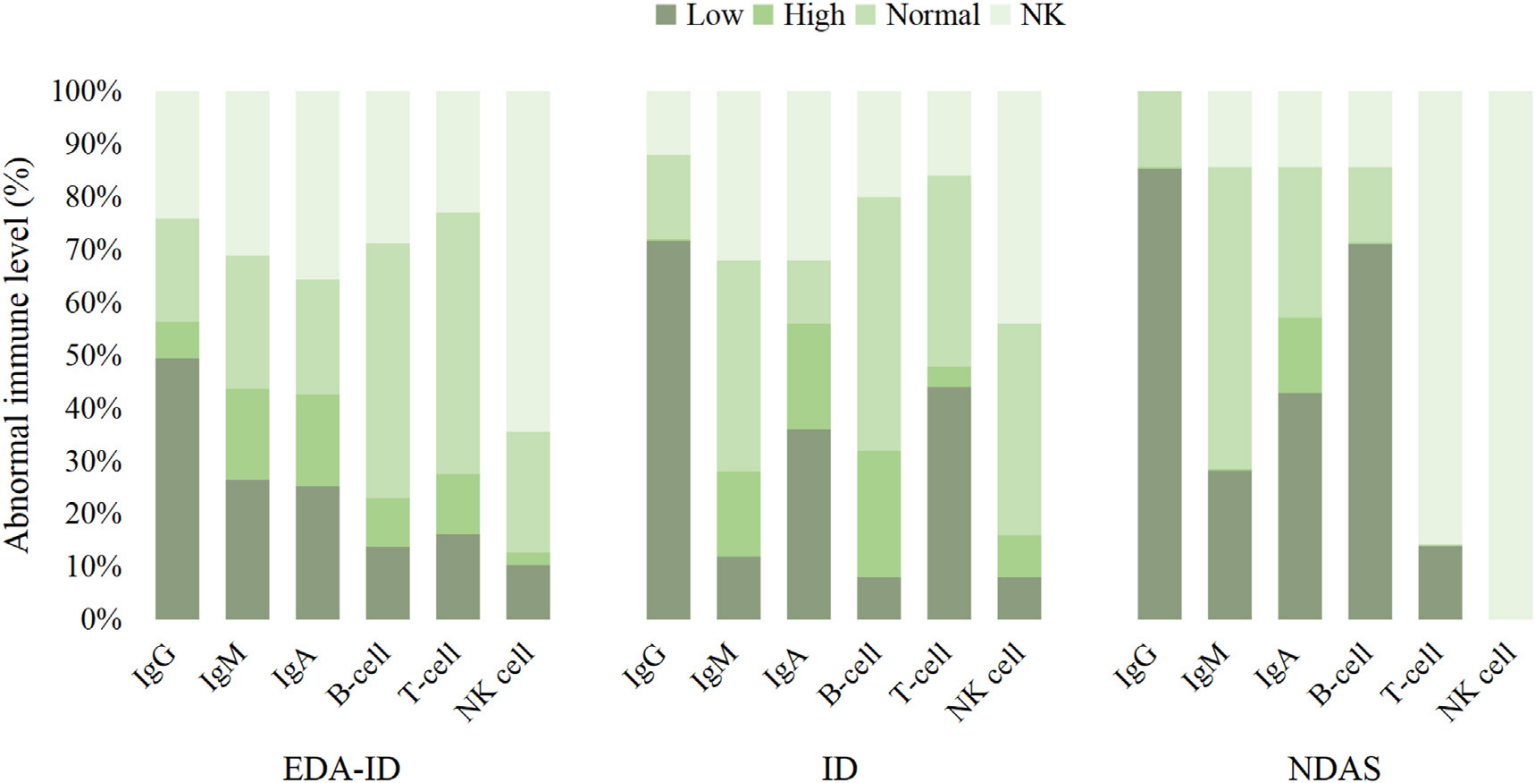
Immune status of EDA-ID, ID, and NDAS patients with *NEMO* mutation. NEMO, nuclear factor-κB essential modulator; EDA-ID, anhidrotic ectodermal dysplasia with immunodeficiency; ID, immunodeficiency; NDAS, *IKBKG* deleted exon 5 autoinflammatory syndrome.

The primary consequences of immunological dysfunction due to *IKBKG* mutations were EDA-ID, ID, and NDAS. The most common manifestation of IP was an increase in the eosinophil count. Antibody deficiencies were prominent in patients with EDA-ID. Approximately half of the patients had hypogammaglobulinemia, and one-third had low levels of IgM and IgA. However, most patients had normal B-cell levels. T-cell abnormalities were occasionally observed, whereas abnormalities in natural killer cells are extremely rare. Patients with ID exhibited similar issues. Additionally, our data support the finding that in patients with EDA-ID, 90.9% of those with hyper-IgM syndrome had hypogammaglobulinemia. Moreover, patients with NDAS were found to exhibit abnormal antibody levels, with decreased B-cell counts, distinguishing them from patients with EDA-ID or ID (Fig. 4).

**Figure 5:**
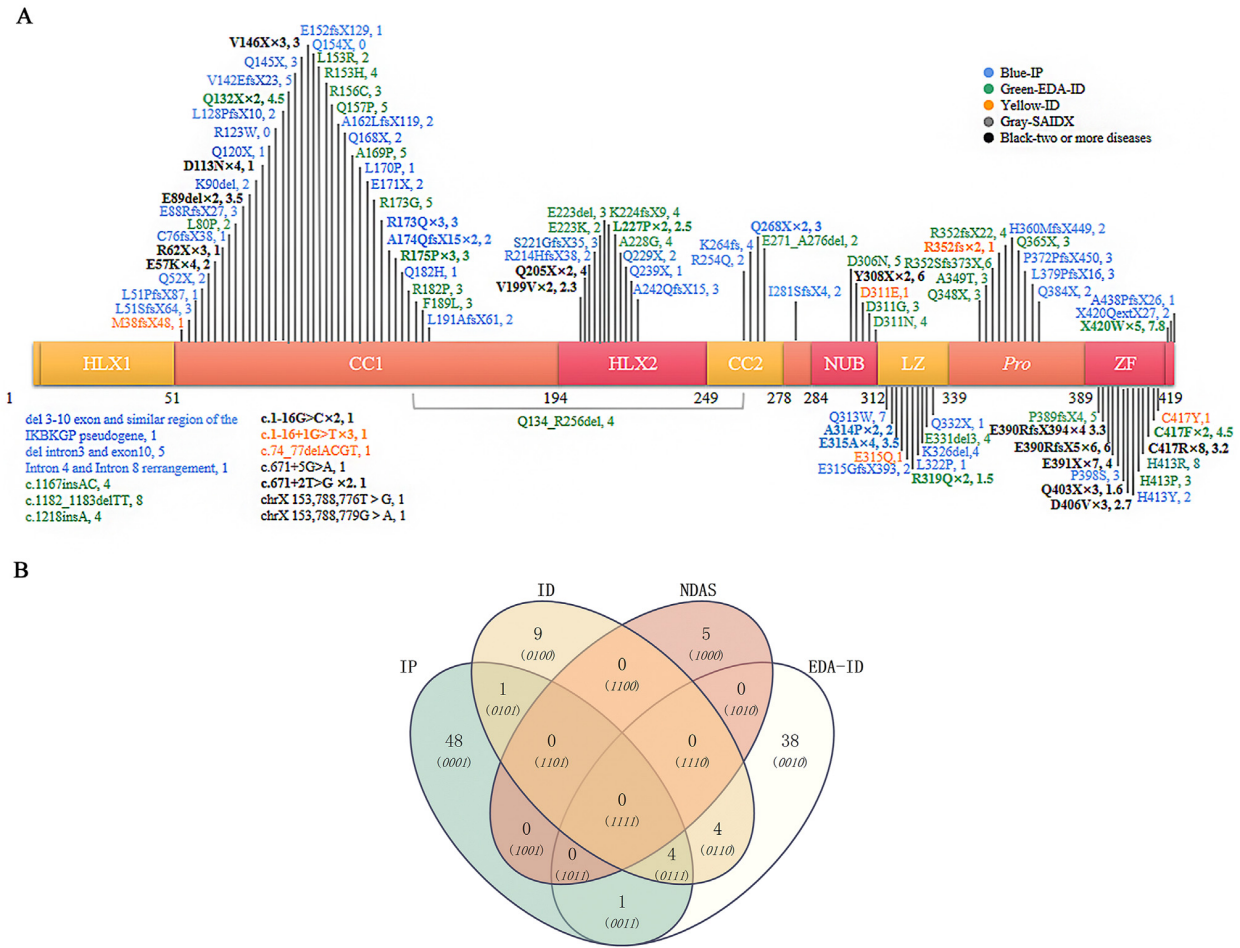
Locations and distribution of *NEMO* gene mutations in IP, ID, EDA-ID, and NDAS. **(A)** Schematic diagram of *NEMO* mutation sites. IP-related mutations are highlighted in blue, EDA-ID-related mutations are highlighted in green, ID without EDA are highlighted in yellow, and black represents two or more diseases caused by one mutation site. The lower left quadrant indicates nucleotide mutations for which the impact on protein alterations is yet to be determined. **(B)** Venn diagram of common mutations in different diseases in patients with *NEMO* mutations. NEMO, nuclear factor-κB essential modulator; IP, incontinentia pigmenti; EDA-ID, anhidrotic ectodermal dysplasia with immunodeficiency; ID, immunodeficiency; NDAS, *IKBKG* deleted exon 5 autoinflammatory syndrome; HLX, HeLical domain (HLX1/2); CC1/2, coiled-coil motif 1/2; NUB, *NEMO* ubiquitin binding domain, LZ, leucine zipper domain; Pro, proline-rich region; ZF, zinc finger.

Del4-10 is the most prevalent type of *IKBKG* mutation. This systematic review encompassed 112 other mutations, as depicted in Figure 5A, illustrating mutation locations and corresponding clinical scores. Each clinical manifestation in the system was assigned a score of 1 point, with an additional point for inflammation. Mutations related to IP (except for exon 4–10 deletion) are concentrated in the CC1 domain and are characterized by the mildest clinical symptoms. IP had the highest clinical manifestation score in the LZ region, followed by the zinc finger (ZF) region. Mutations related to EDA-ID were concentrated in the ZF region and were characterized by severe clinical symptoms. The number of EDA-ID mutations in the CC1 region was relatively large and severe. ID mutations were also concentrated in the ZF region; most had the same gene mutations as those in EDA-ID. NDAS focused on exon 5 mutations such as c.1182_1183delTT, H413R, X420W, Q313W, R153H, Q205X, and Y308X, which could cause clinical manifestations in at least six organ systems. c.1167_1168insC, apart from del4-10, is the most common mutation. E390RfsX5 can cause IP as well as EDA-ID and ID. Table 2 lists some special gene mutations' detailed information and clinical features, including gene sites with many mutations, multiple clinical manifestations, and those associated with special diseases or syndromes; the references cited in Table 2 are also listed here.^4^^,^^8^^,^^12^,^15−67^ Figure 5B depicts the interplay between various mutations in *IKBKG* across different diseases.

**Table 2:** Detailed clinical information of some special *NEMO* mutation.

Nucleotide change(n)		Amino acid change	Mutation type	Exon involved	Phenotype score	Disease	Clinical features									References
							CNS	Eye s	Hair	Teeth	Palate	Breas t	Nai ls	Skin	Others	
Mosaics	Dup from Intron3 to Exon6	K224fsX9	Duplication	From Intron3 to Exon6	Min = 1; Max = 5; Mean = 2.8	IP; EDA-ID; NDAS	Y	Y	–	Y	–	–	Y	Y	Tuner syndrome	Maingay-de Groof et al (2008)^42^
	–	–	–	–											Facial asymmetry	Rashidghamat et al (2016)^59^
	c. 671 + 3 G> C	–	Intronic mutation	Exon 5											Neutrophilic dermatitis; extensive panniculitis; fat necrosis; autoinflammatory syndromes	Hegazy et al (2022)^25^
	–	R175P R182P L227P Q348X	–	Exon 5 Exon 6 Exon 6 Exon 9											IBD	Kawai et al (2012)^35^
c.1167_1168insC		E390fsX394 E390RfsX5	Frameshift; Frameshift and early termination	Exon 10	Min = 1; Max = 6; Mean = 3.3	IP, EDA-ID-OL, ID	–	Y	Y	Y	–	–	–	Y	Infections	Zonana et al (2000)^67^ Orange et al (2004)^51^
															Pulmonary hypertension	Ohnishi et al (2017)^49^
															GVHD	Martuszewski et al (2020)^45^
															Tufting enteropathy; osteosclerosis; lymphedema	Permaul et al (2009)^55^
															Hemophagocytic disease	Schmid et al (2006)^62^
															Hyper IgM syndrome	Zonana et al (2000)^67^
															Anhidrosis	Chang et al (2008)^18^
c.169G > A		E57K	Missense	Exon 2	Min = 0; Max = 4 Mean = 2	IP, EDA-ID	–	–	Y	Y	Y	–	Y	Y	Infections Cutaneous syndactyly	Keller et al (2011)^36^ Pengelly et al (2015)^54^
c.1217A > T		D406V	Missense	Exon 10	Min = 2; Max = 4; Mean = 2.7	IP, EDA-ID	–	–	–	–	–	–	–	Y	Infections; anhidrosis; Behcet's disease; hyper IgM syndrome	Takada et al (2010)^65^ Jain et al (2001)^34^
c.337G > A		D113N	Missense	Exon 3	Min = 1; Max = 1 Mean = 1	EDA-ID; ID	–	–	–	–	–	–	–	–	Infections	Keller et al (2011)^36^ Heller et al (2020)^26^
c.1171G > T		E391X	Nonsense	Exon 10	Min = 1; Max = 4 Mean = 1.8	EDA-ID; ID	–	–	–	Y	–	–	–	–	Infections	Zonana et al (2000)^67^ Cheng et al (2009)^19^
c.1207C > T		Q403X	Nonsense	Exon 10	Min = 2; Max = 2 Mean = 1.6	EDA-ID, ID	–	–	–	–	–	–	–	Y	Infections; kidney abscess;	Huppmann et al (2015)^29^ Orange et al (2004)^51^
c.1249T > C		C417R	Missense	Exon 10	Min = 1; Max = 6; Mean = 3.2	EDA-ID; ID	–	–	Y	Y	–	–		Y	Infections	Orange et al (2004)^51^
															Bronchiectasis; Pulmonary insufficiency; Hyper IgM syndrome	Zonana et al (2000)^67^ Jain et al (2001)^34^
															Anhidrosis	Orange et al (2002)^50^
Del 4-10		–	Deletion	Exon 4-10	Min = 0; Max = 10; Mean = 2.5	IP	Y	Y	Y	Y	Y	Y	Y	Y	Infection	Ogasawara et al (2019)^48^ Pauly et al (2005)^53^
															Pilocytic astrocytoma	Bayart et al (2018)^16^
															Deformity (foot; finger; face)	Kmetz et al (2009)^38^ Fusco et al (2017)^22^ Guevara et al (2016)^24^
															Blind; Franceschetti-Jadassohn's Syndrome	Gregersen et al (2013)^23^
															Bipolar disorder; subungual tumors	Kibbi et al (2018)^37^
															West syndrome	Margari et al (2013)^4^
															SLE; dysmorphic hands; pes planovalgus	Piccoli et al (2012)^56^
															Conversion disorder	Wang et al (2013)^66^
															Psychomoter retardation	Hsiao et al (2010)^27^
															Pulmonary hypertension	Alshenqiti et al (2017)^15^
															Blind; dilated veins	Salamon et al (2016)^63^
															Wilms' tumor	Mariath et al (2018)^44^
Del 4–10 (XXY)		–	Del 4–10 47, XXY	Exon 4-10	Min = 2; Max = 7 Mean = 4.5	IP	Y	Y	Y	–	–	–	–	Y	Klinefelter syndrome; aplasia cutis congenita; difficulty swallow	Moro et al (2020)^47^
c.172_173delAA		N58SfsTer	Deletion mutation and early termination	Exon 2	5	IP	–	Y	Y	Y	–	–	Y	Y	Infection	Ergin et al (2022)^21^
c.425_426delGT		V142EfsX23	Frameshift	Exon 4	5	IP	–	Y	Y	Y	–	–	–	Y	Ectatic and malformed portal tracts; bile plugs; hypoplasia of portal vein radicles	Danescu et al (2018)^28^
Del intron3 and exon10		–	Deletion mutation	Intron 3 exon 10	5	IP	Y	Y	–	–	–	–	–	Y	Progressive proximal skeletal muscle weakness; dilatative cardiomyopathy	Huttner et al (2010)^30^
c.262_264delGAG		E89del	Frame deletion	Exon 3	6	EDA-ID	–	–	Y	Y	–	–	–	Y	Infections; hyper IgM syndrome; anhidrosis	Inaba et al (2021)^32^
c.437T > G		V146G	Missense	Exon 4	Min = 2; Max = 4 Mean = 4	EDA-ID	Y	–	–	Y	–	–	–	–	Infections; generalized lymphadenopathy	Devora et al (2010)^20^
c.505G > C		A169P	Missense	Exon 4	5	EDA-ID	–	–	–	Y	–	–	–	Y	Infections; Crohn's disease; anhidrosis	Mizukami et al (2012)^46^
c.518C > G		R173G	Missense	Exon 4	5	EDA-ID	–	–	Y	Y	–	–	–	Y	Infections; anhidrosis	Ku et al (2007)^40^
c.613C > T		Q205X	Missense	Exon 5	6	EDA-ID	–	–	Y	Y	–	–	–	Y	Infections; GVHD	Yilmaz et al (2022)^64^
c.761G > A		R254Q	Missense	Exon 6	2	EDA-ID	–	–	–	–	–	–	–	Y	AIHA	Huppmann et al (2015)^29^
c.916G > A		D306N	Missense	Exon 10	5	EDA-ID	–	–	–	Y	–	–	–	Y	Pulmonary tuberculosis; ITP;	Ramírez-Alejo et al (2015)^58^
c.923A > G		Y308C	Missense	Exon 4	Min = 4; Max = 8 Mean = 6	EDA-ID	–	–	Y	Y	–	–	–	Y	Infections; psoriasis; atopic dermatitis; cutaneous sarcoidosis	Kolitz et al (2021)^39^
c.944A > C		E315A	Missense	Exon 8	Min = 2; Max = 5; Mean = 3.5	EDA-ID	–	–	Y	Y	–	–	–	Y	Infections; deafness; agenesis corpus callosum and right kidney	Huppmann et al (2015)^29^
c.1027+5G > A		–	Splice mutation	A skipping of exon 4, exon 5, and exon 6	Min = 2; Max = 6 Mean = 4	EDA-ID	–	–	Y	Y	–	–	–	–	Infections; protein-losing enteropathy; pulmonary embolization; Crohn's disease; infant death syndrome	Ørstavik et al (2006)^52^
c.1117+1G > A		R352Sfs373X	Frameshift and early termination	Exon 9	6	EDA-ID	–	–	–	–	–	–	–	–	Infections; lymphedema; hepatic veno-occlusive disease/sinusoidal obstruction syndrome; autoimmune thrombocytopenia; GVHD;	Heller et al (2020)^26^
c.1167dupC		E390RfsX5	Frameshift	Exon 10	Min = 2; Max = 7 Mean = 5.1	EDA-ID	–	–	Y	Y	–	–	–	Y	Infections; anemia; SCID; GVHD; skull deformity; chronic diarrhea; septic shock; anhidrosis; eczema;	Martuszewski et al (2020)^45^
c.1250G > T		C417F	Missense	Exon 10	Min = 4; Max = 5 Mean = 4.5	EDA-ID	–	–	Y	Y	–	–	–	Y	Infections; anhidrosis;	Zonana et al (2000)^67^
c.458G > A		R153H	Missense	Exon 3	6	EDA-ID-OL	–	–	Y	Y	–	–	–	–	Infections; lymphedema; osteoporosis; anhidrosis	Keller et al (2011)^36^
c.470A > C		Q157P	Missense	Exon 4	5	EDA-ID-OL	–	–	Y	–	–	–	–	Y	Infections; dysmorphic; osteopetrosis	Carlberg et al (2014)^17^
c.1182_1183delTT		–	Frameshift	Exon 10	8	EDA-ID-OL	Y	–	Y	Y	–	–	–	Y	Infections; lymphoedema; osteopetrosis; asthma; hypothytoidism; anhidrosis	Roberts et al (2010)^61^
c.1238A > G		H413R	Missense	Exon 10	8	EDA-ID-OL	Y	–	Y	Y	–	–	–	Y	Infections; lymphoedema; osteopetrosis; saddle nose; frontal bossing; growth retardation. MAS	Silvia et al (2017)^60^
c.1259A > G		X420W	Stop lost	Exon 10	Min = 5; Max = 10 Mean = 7.8	EDA-ID-OL	Y	Y	Y	Y	–	–	–	Y	Anhidrosis; osteosclerosis; lymphedema; sepsis; capillary hemangiomas; anemia; thrombocytopenia; intestinal obstruction; multiple lymphangiomata; gastroenteritis;	Mansour et al (2001)^43^ Döffinger, et al (2001)^8^
c.185G > A		R62Q	Missense	Exon 2	Min = 1; Max = 2 Mean = 1.3	ID	–	–	–	–	–	–	–	–	Infections; lymphocytic colitis; granulomatous Hepatitis; autoimmune hemolytic anemia; immune thrombocytopenia; Evans syndrome	Rae et al (2017)^57^
c.931C > G		D311E	Missense	Exon 8	4	ID	–	–	–	–	–	–	–	–	Nasopharyngeal carcinoma; disseminated BCG infection; cervical lymphadenitis; parotitis; sepsis	Imamura et al (2011)^31^
c.943G > C		E315Q	Missense	Exon 8	5	ID	–	–	–	–	–	–	–	–	Lymph node tuberculosis; sepsis; squamous cell carcinoma; thyroid papillary carcinoma; langerhans cell histiocytosis	Inoue et al (2018)^33^
c.597G > A		V199V	Synonymous	Exon 5	Min = 2; Max = 3; Mean = 2.3	NDAS	Y	Y	–	Y	–	–	–	Y	Infections; sterile panniculitis; optic neuritis; chorioretinitis; granulomatous panniculitis;	Lee et al (2022)^41^
															Granulomatous uveitis; granulomatous hepatitis	de Jesus et al (2020)^12^
ChrX 153,788,776T > G		–	Splice mutation	Exon 5	4	NDAS	–	–	–	–	–	–	–	–	Lymphohistiocytic panniculitis; anemia; G6PD deficiency; thrombocytopenia; CNS bleed; cortical atrophy	Lee et al (2022)^41^

Y: yes, —: no, CNS: central nervous system, SLE: systematic lupus erythematosus, SCID: severe combined immune deficiency, GVHD: graft versus host disease, IBD: inflammatory bowel disease, AIHA: autoimmune hemolytic anemia, MSA: macrophage activation syndrome, ITP: immune thrombocytopenic purpura, G6PD: glucose 6 phosphate dehydrogenase.

## Discussion

The diverse clinical presentations of *IKBKG* gene mutations are fascinating and warrant further exploration. The diagnosis of IP and EDA-ID necessitates a comprehensive evaluation of diverse clinical presentations and laboratory findings, alongside differential diagnoses from related conditions. Our comparative analysis in Figure 2 delineates the clinical manifestations associated with *IKBKG* related disorders. Notably, central nervous system abnormalities manifest at a prevalence of approximately 40 % among IP patients, surpassing the secondary diagnostic criterion of nail anomalies. This suggests the significant role of central nervous system abnormalities in IP, despite central nervous system abnormalities not being included in either the primary or secondary diagnostic criteria for IP. Therefore, our findings strongly support the proposal by Minić et al^68^ to incorporate CNS abnormalities into the diagnostic criteria for IP, cautioning against potential underdiagnosis and delayed diagnosis in the absence of such inclusion. *IKBKG* mutations exhibit marked heterogeneity in sex selection and phenotypes. For instance, V146G can manifest as IP in females,^69^ while present as EDA-ID in males.^20^ Historically, mutations in the N-terminal region of *IKBKG* were linked with female IP, while those in the C-terminal region correlated with male EDA-ID.^17^ However, our study suggests that this paradigm may not universally apply. As illustrated in Figure 5A, mutations associated with IP and EDA-ID are dispersed across various structural domains of *IKBKG*, indicating a complex genotype–phenotype relationship. Nevertheless, Figure 5A highlights that mutations occurring in the ZF domain contribute to greater phenotypic diversity, harboring a multitude of variants capable of causing multiple disorders. For example, Y308X and C417R can exhibit as EDA-ID in some individuals, whereas in others, they may solely manifest as ID.^39^^,^^50^^,^^51^^,^^70^ E390RfsX5 can even cause not only IP as well as EDA-ID and ID.^49^^,^^71^^,^^72^ Furthermore, mutations occurring here also imply more severe manifestations. Stephanie et al^26^ mentioned that mutations in the ZF structural domain are often complex and life-threatening. This is consistent with our research findings. Certain mutations, such as X420W, H413R, Q157P, E390RfsX5, and c.1182_1183delTT, induce premature stop codons, leading to OL-EDA-ID, the most severe clinical phenotype induced by hypomorphic mutations in *IKBKG*.^8^^,^^17^^,^^55^^,^^60^^,^^61^ Among the observed mutations, X420W induced the most severe manifestations. This is attributed to its location as a codon within the *IKBKG* gene, which markedly influences the bioactivity of *IKBKG* and the operational efficiency of the NF-κB signaling pathway.^73^ We reviewed mutations in the ZF structural domain and found that most cases were EDA-ID.^67^^,^^74^ Furthermore, considering the severity of EDA-ID and ID associated with mutations in the ZF region, there is a propensity for immune-related complications. Mutations in the ZF region can impair NF-κB pathway activation in immune cells, compromising host defense mechanisms against microbial pathogens — a primary factor contributing to mortality in patients with *IKBKG* mutations.^75^ However, our correlation analysis between mutation sites and clinical severity did not reveal significant differences (*P* > 0.05). This may be related to an insufficient sample size.

Since NF-κB is typically associated with the development of tumors, such as small cell lung cancer, rectal cancer, and multiple myeloma,^76^, ^77^, ^78^,^76^, ^77^, ^78^ some types of *IKBKG* mutations accompany tumors. For instance, the del4-10 mutation leads to IP combined with Wilms' tumor,^44^ the X420W mutation leads to OL-EDA-ID combined with lymphangioma,^43^ the D311E mutation leads to ID combined with nasopharyngeal carcinoma,^31^ and the E315Q mutation leads to ID combined with squamous cell carcinoma of the skin and thyroid cancer.^33^ The correlation between these mutations and tumor development is unknown; however, vigilance should be increased.

The immunological abnormalities caused by *IKBKG* mutations are particularly prominent. Our data indicate that immunological abnormalities in patients with EDA-ID, ID, and NDAS are primarily antibody deficiencies, predominantly IgG deficiency; varying levels of abnormalities in IgM and IgA antibodies may accompany them. In EDA-ID, elevated levels of IgM can indicate hyper-IgM syndrome, predisposing individuals to opportunistic infections.^34^ Therefore, this should be carefully monitored. In our study, a majority of EDA-ID cases accompanied by hyper-IgM syndrome occurred in the ZF region and few cases of EDA-ID children with hyper-IgM syndrome were caused by mutations located in other regions.^79^^,^^80^ The main reason is that the ZF structural domain disrupts the degradation of the NF-κB inhibitor IκB-α mediated by CD40L, thereby affecting the class switch of immunoglobulins,^34^ and there is a noted correlation with *Pneumocystis jirovecii* infection. Another notable immunological anomaly observed in EDA-ID patients is polysaccharide antibody deficiency. Unfortunately, our study did not comprehensively gather data on this immune aberration, which can occasionally manifest as the sole laboratory anomaly in EDA-ID patients, especially when concurrent with dental anomalies.^81^ Dental abnormalities are hallmark features of ectodermal dysplasia, with tooth anomalies being the predominant developmental irregularity observed in EDA-ID associated with *IKBKG* mutations. In physiological conditions, activation of the NF-κB pathway downstream of the ectodysplasin, edar, and edar-associated death domain plays a crucial role in odontogenesis and other ectodermal developmental processes. Dysfunctions in ectodysplasin, edar, or edar-associated death domain can precipitate ectodermal dysplasia. As a downstream regulator within this pathway, *IKBKG* mutations can also induce ectodermal dysplasia, particularly affecting tooth tip formation.^82^ Therefore, *IKBKG* mutations often lead to cone-shaped teeth.

In this study, the number of B-cells in most patients with EDA-ID and ID was usually normal, and T-cells and natural killer cells were relatively less affected. In NDAS, more than half of the patients have a low number of B cells, suggesting the need for further fine lymphocyte phenotyping of patients with *IKBKG* mutations. Notably, when *IKBKG* affects T cells, it may coincide with the onset of inflammatory bowel disease, such as somatic mosaicism involving R175P, R182P, L227P, and Q348X^35^ in X-linked anhidrotic ectodermal dysplasia with immunodeficiency frequently lower NF-κB activity, thereby impacting antigen presentation, T cell differentiation, and function, leading to significant T cell infiltration of the intestinal wall.^19^ The influence of *IKBKG* mutations on Toll-like receptor (TLR) signaling pathways is often cited to explain the susceptibility of *IKBKG* patients to various pathogens. Impairments in TLR4 response to lipopolysaccharide and partially compromised CD40 signaling may relate to infections with gram-negative bacteria and *Pneumocystis carinii*. IL-1b, IL-18, TLR2, or other NF-kB-dependent signaling pathways' diminished responses may be associated with infections from gram-positive bacteria. Severe mycobacterial infections may also be linked to impaired TLR signaling. Most patients with IP exhibit eosinophilia, most noticeable in stages 1 and 2. These abnormalities can be detected in the periphery and the bone marrow, ranging from 5% to 79%. This mechanism is related to the overactivation of keratinocytes.^83^ Over time, most children eventually normalized, highlighting the importance of aiding the early diagnosis of IP.

In terms of treatment, early infection control is important because severe infections are often the main cause of death in patients with *IKBKG* mutations, especially in EDA-ID patients with impaired C-reacting protein responses. According to our research, *Mycobacteria*, *Streptococcus*, *Staphylococcus*, and *Escherichia coli* are the most common pathogens that should be monitored in children with suspected *IKBKG* mutations. If conventional anti-infective therapy is ineffective, several other pathogens must be considered, such as MRSA, *Klebsiella*, *Enterococcus faecalis*, *Haemophilus influenzae*, and *Pseudomonas aeruginosa*. Alternative intravenous immunoglobulin therapy is available for patients with impaired immunoglobulin conversion.^7^ Hematopoietic stem-cell transplantation is usually used in situations that pose a severe threat to life or in cases presenting with mesodermal defects of *IKBKG*,^26^^,^^84^ such as OL-EDA-ID.^7^ The global survival rate after hematopoietic stem-cell transplantation among *IKBKG*-deficient children was 74% at a median follow-up after hematopoietic stem-cell transplantation of 57 months, and most of the clinical symptoms caused by *IKBKG* mutation were curable, except for colitis.^84^ Some targeted treatments, such as NEMO-binding domain peptide and CRISPR/Cas9, and therapies involving embryonic stem cells, or induced pluripotent stem cells, are currently under research and development.^85^

Clinical diseases caused by *IKBKG* gene mutations are generally still in the early stages of exploration and require more attention. Moreover, newly discovered disease subtypes, such as NDAS, require further sharing and discussion regarding their diagnosis and treatment. We look forward to further discoveries regarding *IKBKG* mutations.

## CRediT authorship contribution statement

**Jin Wang:** Data curation, Formal analysis, Writing – original draft. **Kexin Shen:** Data curation, Visualization. **Hongxia Lou:** Software. **Lina Zhou:** Methodology, Resources. **Yunfei An:** Supervision. **Xiaodong Zhao:** Supervision, Writing – review & editing. **Yuan Ding:** Conceptualization, Supervision, Writing – review & editing.

## Conflict of interests

Xiaodong Zhao is a deputy editor-in-chief for *Genes & Diseases* and was not involved in the editorial review or the decision to publish this article. The other authors declared no competing interests.

## References

[1] Hayden M.S., Ghosh S. (2008). Shared principles in NF-kappaB signaling. Cell.

[2] Senegas A., Gautheron J., Maurin A.G., Courtois G. (2015). IKK-related genetic diseases: probing NF-κB functions in humans and other matters. Cell Mol Life Sci.

[3] Wang R., Lara-Corrales I., Kannu P., Pope E. (2019). Unraveling incontinentia pigmenti: a comparison of phenotype and genotype variants. J Am Acad Dermatol.

[4] Margari L., Lamanna A.L., Buttiglione M., Craig F., Petruzzelli M.G., Terenzio V. (2013). Long-term follow-up of neurological manifestations in a boy with incontinentia pigmenti. Eur J Pediatr.

[5] Smahi A., Courtois G., Rabia S.H. (2002). The NF-kappaB signalling pathway in human diseases: from incontinentia pigmenti to ectodermal dysplasias and immune-deficiency syndromes. Hum Mol Genet.

[6] Smahi A., Courtois G., Vabres P. (2000). Genomic rearrangement in *NEMO impairs* NF-kappaB activation and is a cause of incontinentia pigmenti. The International Incontinentia Pigmenti (IP) Consortium. Nature.

[7] Kawai T., Nishikomori R., Heike T. (2012). Diagnosis and treatment in anhidrotic ectodermal dysplasia with immunodeficiency. Allergol Int.

[8] Döffinger R., Smahi A., Bessia C. (2001). X-linked anhidrotic ectodermal dysplasia with immunodeficiency is caused by impaired NF-kappaB signaling. Nat Genet.

[9] Jimi E., Katagiri T. (2022). Critical roles of NF-κB signaling molecules in bone metabolism revealed by genetic mutations in osteopetrosis. Int J Mol Sci.

[10] Swarnkar G., Shim K., Nasir A.M. (2016). Myeloid deletion of *Nemo* causes osteopetrosis in mice owing to upregulation of transcriptional repressors. Sci Rep.

[11] Frans G., van der Werff Ten Bosch J., Moens L. (2017). Functional evaluation of an IKBKG variant suspected to cause immunodeficiency without ectodermal dysplasia. J Clin Immunol.

[12] de Jesus A.A., Hou Y., Brooks S. (2020). Distinct interferon signatures and cytokine patterns define additional systemic autoinflammatory diseases. J Clin Invest.

[13] Page M.J., McKenzie J.E., Bossuyt P.M. (2021). The PRISMA 2020 statement: an updated guideline for reporting systematic reviews. J Clin Epidemiol.

[14] Murad M.H., Sultan S., Haffar S., Bazerbachi F. (2018). Methodological quality and synthesis of case series and case reports. BMJ Evid Based Med.

[15] Alshenqiti A., Nashabat M., AlGhoraibi H., Tamimi O., Alfadhel M. (2017). Pulmonary hypertension and vasculopathy in incontinentia pigmenti: a case report. Therapeut Clin Risk Manag.

[16] Bayart C.B., Ishak G.E., Finn L.S. (2018). Pilocytic astrocytoma with leptomeningeal spread in a patient with incontinentia pigmenti presenting with unilateral nystagmus. Pediatr Blood Cancer.

[17] Carlberg V.M., Lofgren S.M., Mann J.A. (2014). Hypohidrotic ectodermal dysplasia, osteopetrosis, lymphedema, and immunodeficiency in an infant with multiple opportunistic infections. Pediatr Dermatol.

[18] Chang T.T., Behshad R., Brodell R.T., Gilliam A.C. (2008). A male infant with anhidrotic ectodermal dysplasia/immunodeficiency accompanied by incontinentia pigmenti and a mutation in the *NEMO* pathway. J Am Acad Dermatol.

[19] Cheng L.E., Kanwar B., Tcheurekdjian H. (2009). Persistent systemic inflammation and atypical enterocolitis in patients with *NEMO* syndrome. Clin Immunol.

[20] Devora G.A., Sun L., Chen Z. (2010). A novel missense mutation in the nuclear factor-κB essential modulator (*NEMO*) gene resulting in impaired activation of the NF-κB pathway and a unique clinical phenotype presenting as MRSA subdural empyema. J Clin Immunol.

[21] Ergin F.B.C., Tekin M., Güneş M., Güneş B., Baysun Ş., Akar N. (2022). A Turkish case of incontinentia pigmenti with a deletion mutation at inhibitor of kappa B kinase gamma gene. Egypt J Med Hum Genet.

[22] Fusco F., Conte M.I., Diociaiuti A. (2017). Unusual father-to-daughter transmission of incontinentia pigmenti due to mosaicism in IP males. Pediatrics.

[23] Gregersen P.A., Sommerlund M., Ramsing M., Gjørup H., Rasmussen A.A., Aggerholm A. (2013). Diagnostic and molecular genetic challenges in male incontinentia pigmenti: a case report. Acta Derm Venereol.

[24] Guevara B.E.K., Hsu C.K., Liu L. (2016). Improved molecular diagnosis of the common recurrent intragenic deletion mutation in IKBKG in a Filipino family with incontinentia pigmenti. Australas J Dermatol.

[25] Hegazy S., Marques M.C., Canna S.W. (2022). *NEMO*-NDAS: a panniculitis in the young representing an autoinflammatory disorder in disguise. Am J Dermatopathol.

[26] Heller S., Kölsch U., Magg T. (2020). T cell impairment is predictive for a severe clinical course in *NEMO* deficiency. J Clin Immunol.

[27] Hsiao P.F., Lin S.P., Chiang S.S., Wu Y.H., Chen H.C., Lin Y.C. (2010). NEMO gene mutations in Chinese patients with incontinentia pigmenti. J Formos Med Assoc.

[28] Hull S., Arno G., Thomson P. (2015). Somatic mosaicism of a novel IKBKG mutation in a male patient with incontinentia pigmenti. Am J Med Genet A.

[29] Huppmann A.R., Leiding J.W., Hsu A.P. (2015). Pathologic findings in *NEMO deficiency:* a surgical and autopsy survey. Pediatr Dev Pathol.

[30] Huttner H.B., Richter G., Jünemann A. (2010). Incontinetia pigmenti-related myopathy or unsolved "double trouble"?. Neuromuscul Disord.

[31] Imamura M., Kawai T., Okada S. (2011). Disseminated BCG infection mimicking metastatic nasopharyngeal carcinoma in an immunodeficient child with a novel hypomorphic *NEMO* mutation. J Clin Immunol.

[32] Inaba S., Aizawa Y., Miwa Y. (2021). Case report: analysis of preserved umbilical cord clarified X-linked anhidrotic ectodermal dysplasia with immunodeficiency in deceased, undiagnosed uncles. Front Immunol.

[33] Inoue Y., Shimizu A., Suto M. (2018). Cutaneous squamous cell carcinoma, thyroid cancer and Langerhans cell histiocytosis in a patient with X-linked recessive Mendelian susceptibility to mycobacterial diseases with a nuclear factor-κB essential modifier mutation. J Dermatol.

[34] Jain A., Ma C.A., Liu S., Brown M., Cohen J., Strober W. (2001). Specific missense mutations in *NEMO* result in hyper-IgM syndrome with hypohydrotic ectodermal dysplasia. Nat Immunol.

[35] Kawai T., Nishikomori R., Izawa K. (2012). Frequent somatic mosaicism of *NEMO* in T cells of patients with X-linked anhidrotic ectodermal dysplasia with immunodeficiency. Blood.

[36] Keller M.D., Petersen M., Ong P. (2011). Hypohidrotic ectodermal dysplasia and immunodeficiency with coincident *NEMO* and EDA mutations. Front Immunol.

[37] Kibbi N., Totonchy M., Suozzi K.C., Ko C.J., Odell I.D. (2018). A case of subungual tumors of incontinentia pigmenti: a rare manifestation and association with bipolar disease. JAAD Case Rep.

[38] Kmetz E.C., Pai G.S., Burges G.E. (2009). Incontinentia pigmenti with a foreshortened hand: evidence for the significance of NFkappaB in human morphogenesis. Pediatr Dermatol.

[39] Kolitz E., Chamseddin B., Son R. (2021). A novel *NEMO/IKBKG* mutation identified in a primary immunodeficiency disorder with recurrent atypical mycobacterial infections. JAAD Case Rep.

[40] Ku C.L., Picard C., Erdös M. (2007). IRAK4 and *NEMO* mutations in otherwise healthy children with recurrent invasive pneumococcal disease. J Med Genet.

[41] Lee Y., Wessel A.W., Xu J. (2022). Genetically programmed alternative splicing of *NEMO mediates* an autoinflammatory disease phenotype. J Clin Invest.

[42] Maingay-de Groof F., Lequin M.H., Roofthooft D.W. (2008). Extensive cerebral infarction in the newborn due to incontinentia pigmenti. Eur J Paediatr Neurol.

[43] Mansour S., Woffendin H., Mitton S. (2001). Incontinentia pigmenti in a surviving male is accompanied by hypohidrotic ectodermal dysplasia and recurrent infection. Am J Med Genet.

[44] Mariath L.M., Santa Maria F.D., Poziomczyk C.S. (2018). Intrafamilial clinical variability in four families with incontinentia pigmenti. Am J Med Genet A.

[45] Martuszewski A., Paluszkiewicz P., Sierżęga-Staykov K. (2020). Successful allogeneic stem cell transplantation in nuclear factor-kappa B essential modulator deficiency syndrome after treosulfan-based conditioning: a case report. Transplant Proc.

[46] Mizukami T., Obara M., Nishikomori R. (2012). Successful treatment with infliximab for inflammatory colitis in a patient with X-linked anhidrotic ectodermal dysplasia with immunodeficiency. J Clin Immunol.

[47] Moro R., Fabiano A., Calzavara-Pinton P. (2020). Incontinentia pigmenti associated with aplasia cutis congenita in a newborn male with klinefelter syndrome: is the severity of neurological involvement linked to skin manifestations?. Dermatol Ther.

[48] Ogasawara K., Honda Y., Maeda H., Sato M., Nakano H., Hosoya M. (2019). Corticosteroid therapy in neonatal incontinentia pigmenti with asymptomatic cerebral lesions. Pediatr Neurol.

[49] Ohnishi H., Kishimoto Y., Taguchi T. (2017). Immunodeficiency in two female patients with incontinentia pigmenti with heterozygous *NEMO* mutation diagnosed by LPS unresponsiveness. J Clin Immunol.

[50] Orange J.S., Brodeur S.R., Jain A. (2002). Deficient natural killer cell cytotoxicity in patients with IKK-gamma/*NEMO* mutations. J Clin Invest.

[51] Orange J.S., Jain A., Ballas Z.K., Schneider L.C., Geha R.S., Bonilla F.A. (2004). The presentation and natural history of immunodeficiency caused by nuclear factor kappaB essential modulator mutation. J Allergy Clin Immunol.

[52] Ørstavik K.H., Kristiansen M., Knudsen G.P. (2006). Novel splicing mutation in the *NEMO* (IKK-gamma) gene with severe immunodeficiency and heterogeneity of X-chromosome inactivation. Am J Med Genet A.

[53] Pauly E., Linderkamp O., Pöschl J. (2005). Incontinentia pigmenti in combination with decreased IgG subclass concentrations in a female newborn. Biol Neonate.

[54] Pengelly R.J., Upstill-Goddard R., Arias L. (2015). Resolving clinical diagnoses for syndromic cleft lip and/or palate phenotypes using whole-exome sequencing. Clin Genet.

[55] Permaul P., Narla A., Hornick J.L., Pai S.Y. (2009). Allogeneic hematopoietic stem cell transplantation for X-linked ectodermal dysplasia and immunodeficiency: case report and review of outcomes. Immunol Res.

[56] Piccoli G.B., Attini R., Vigotti F.N. (2012). *NEMO* syndrome (incontinentia pigmenti) and systemic lupus erythematosus: a new disease association. Lupus.

[57] Rae W., Ward D., Mattocks C.J. (2017). Autoimmunity/inflammation in a monogenic primary immunodeficiency cohort. Clin Transl Immunology.

[58] Ramírez-Alejo N., Alcántara-Montiel J.C., Yamazaki-Nakashimada M. (2015). Novel hypomorphic mutation in IKBKG impairs *NEMO*-ubiquitylation causing ectodermal dysplasia, immunodeficiency, incontinentia pigmenti, and immune thrombocytopenic purpura. Clin Immunol.

[59] Rashidghamat E., Hsu C.K., Nanda A., Liu L., Al-Ajmi H., McGrath J.A. (2016). Incontinentia pigmenti in a father and daughter. Br J Dermatol.

[60] Ricci S., Romano F., Nieddu F., Picard C., Azzari C. (2017). OL-EDA-ID syndrome: a novel hypomorphic *NEMO* mutation associated with a severe clinical presentation and transient HLH. J Clin Immunol.

[61] Roberts C.M., Angus J.E., Leach I.H., McDermott E.M., Walker D.A., Ravenscroft J.C. (2010). A novel *NEMO* gene mutation causing osteopetrosis, lymphoedema, hypohidrotic ectodermal dysplasia and immunodeficiency (OL-HED-ID). Eur J Pediatr.

[62] Pachlopnik Schmid J.M., Junge S.A., Hossle J.P. (2006). Transient hemophagocytosis with deficient cellular cytotoxicity, monoclonal immunoglobulin M gammopathy, increased T-cell numbers, and hypomorphic NEMO mutation. Pediatrics.

[63] Soltirovska Salamon A., Lichtenbelt K., Cowan F.M. (2016). Clinical presentation and spectrum of neuroimaging findings in newborn infants with incontinentia pigmenti. Dev Med Child Neurol.

[64] Surucu Yilmaz N., Bilgic Eltan S., Kayaoglu B. (2022). Low density granulocytes and dysregulated neutrophils driving autoinflammatory manifestations in *NEMO* deficiency. J Clin Immunol.

[65] Takada H., Nomura A., Ishimura M., Ichiyama M., Ohga S., Hara T. (2010). *NEMO* mutation as a cause of familial occurrence of Behçet's disease in female patients. Clin Genet.

[66] Wang Y., Chen Y., Wang Q. (2013). A 14-year-old girl with an unusual combination of incontinentia pigmenti and conversion disorder. Int J Clin Exp Med.

[67] Zonana J., Elder M.E., Schneider L.C. (2000). A novel X-linked disorder of immune deficiency and hypohidrotic ectodermal dysplasia is allelic to incontinentia pigmenti and due to mutations in IKK-gamma (*NEMO*). Am J Hum Genet.

[68] Minić S., Trpinac D., Obradović M. (2014). Incontinentia pigmenti diagnostic criteria update. Clin Genet.

[69] Conte M.I., Pescatore A., Paciolla M. (2014). Insight into IKBKG/*NEMO locus:* report of new mutations and complex genomic rearrangements leading to incontinentia pigmenti disease. Hum Mutat.

[70] Sun S., Li F., Liu Y. (2019). A novel inhibitor of nuclear factor kappa-B kinase subunit gamma mutation identified in an incontinentia pigmenti patient with syndromic tooth agenesis. Arch Oral Biol.

[71] Artac H., Emsen A., Ucaryilmaz H., Emiroglu H.H., Uygun V., Stray-Pedersen A. (2019). Infliximab therapy for inflammatory colitis in an infant with *NEMO* deficiency. Immunol Res.

[72] Alkan G., Artac H., Oz S.K.T., Emiroglu M. (2021). Management of COVID-19 pneumonia in a child with *NEMO* deficiency. Immunol Res.

[73] Fusco F., Pescatore A., Conte M.I. (2015). EDA-ID and IP, two faces of the same coin: how the same IKBKG/*NEMO* mutation affecting the NF-κB pathway can cause immunodeficiency and/or inflammation. Int Rev Immunol.

[74] Khan T.A., Schimke L.F., Amaral E.P. (2016). Interferon-gamma reduces the proliferation of M. tuberculosis within macrophages from a patient with a novel hypomorphic *NEMO* mutation. Pediatr Blood Cancer.

[75] Shifera A.S. (2010). The zinc finger domain of IKKγ (*NEMO*) protein in health and disease. J Cell Mol Med.

[76] Koerner L., Schmiel M., Yang T.P., Peifer M., Buettner R., Pasparakis M. (2023). *NEMO*- and RelA-dependent NF-κB signaling promotes small cell lung cancer. Cell Death Differ.

[77] Yu Z., Gao J., Zhang X. (2022). Characterization of a small-molecule inhibitor targeting *NEMO*/IKKβ to suppress colorectal cancer growth. Signal Transduct Targeted Ther.

[78] Aggarwal B.B. (2004). Nuclear factor-kappaB: the enemy within. Cancer Cell.

[79] Toyohara M., Kajiho Y., Toyofuku E. (2021). An infant with X-linked anhidrotic ectodermal dysplasia with immunodeficiency presenting with *Pneumocystis* pneumonia: a case report. Clin Case Rep.

[80] Aujnarain A., Chung C., Upton J. (2016). Paradoxical hyperhidrosis in a patient with ectodermal dysplasia and immunodeficiency. LymphoSign J.

[81] Gagliardi L., Rusconi F., Bellù R., Zanini R., Italian Neonatal Network (2014). Association of maternal hypertension and chorioamnionitis with preterm outcomes. Pediatrics.

[82] Courtney J.M., Blackburn J., Sharpe P.T. (2005). The ectodysplasin and NFκB signalling pathways in odontogenesis. Arch Oral Biol.

[83] Berlin A.L., Paller A.S., Chan L.S. (2002). Incontinentia pigmenti: a review and update on the molecular basis of pathophysiology. J Am Acad Dermatol.

[84] Miot C., Imai K., Imai C. (2017). Hematopoietic stem cell transplantation in 29 patients hemizygous for hypomorphic *IKBKG/NEMO* mutations. Blood.

[85] Maubach G., Schmädicke A.C., Naumann M. (2017). *NEMO* links nuclear factor-κB to human diseases. Trends Mol Med.

