# Supplementary material for: Clinical relevance of loss-of-function mutations of NEMO/IKBKG
Source: Genes Dis. 2025 Jan 12;12(5):101531. doi: 10.1016/j.gendis.2025.101531 (PMC12221755; doi:10.1016/j.gendis.2025.101531)
Supplement: Multimedia component 1 [file mmc1.docx]

**Supplemental references:**

Abbott, J., Quinones, R., De La Morena, M., & Gelfand, E. (2014). Successful hematopoietic cell transplantation in patients with unique NF-κB essential modulator (NEMO) mutations. *Bone marrow transplantation*, *49*(11), 1446-1447.

Alkan, G., Artac, H., Oz, S. K. T., & Emiroglu, M. (2021). Management of COVID-19 pneumonia in a child with NEMO deficiency. *Immunol Res*, *69*, 391-393.

Alshenqiti, A., Nashabat, M., AlGhoraibi, H., Tamimi, O., & Alfadhel, M. (2017). Pulmonary hypertension and vasculopathy in incontinentia pigmenti: a case report. *Therapeutics and Clinical Risk Management*, 629-634.

Aradhya, S., Courtois, G., Rajkovic, A., Lewis, R. A., Levy, M., Israël, A., & Nelson, D. L. (2001). Atypical forms of incontinentia pigmenti in male individuals result from mutations of a cytosine tract in exon 10 of NEMO (IKK-γ). *The American Journal of Human Genetics*, *68*(3), 765-771.

Ardelean, D., & Pope, E. (2006). Incontinentia pigmenti in boys: a series and review of the literature. *Pediatr Dermatol*, *23*(6), 523-527.

Artac, H., Emsen, A., Ucaryilmaz, H., Emiroglu, H. H., Uygun, V., & Stray-Pedersen, A. (2019). Infliximab therapy for inflammatory colitis in an infant with NEMO deficiency. *Immunol Res*, *67*, 450-453.

Aujnarain, A., Chung, C., & Upton, J. (2016). Paradoxical hyperhidrosis in a patient with ectodermal dysplasia and immunodeficiency. *LymphoSign Journal*, *3*(2), 61-66.

Azarbayjani, Z., Enshaei, Z., & Eshghi, A. (2021). A Report of Incontinentia Pigmenti in an 11-year-old Girl. *Iranian Journal of Pediatrics*, *31*(3).

Azarsiz, E., Karaca, N., Karaca, E., Aksu, G., Genel, F., Gulez, N., Ozen, S., & Kutukculer, N. (2023). Eight years of follow-up experience in children with mendelian susceptibility to mycobacterial disease and review of the literature. *Asian Pacific Journal of Allergy and Immunology*, *41*(4), 372-378.

Bayart, C. B., Ishak, G. E., Finn, L. S., Lee, A., Baran, F., Sun, A., Gupta, D., & Vitanza, N. A. (2018). Pilocytic astrocytoma with leptomeningeal spread in a patient with incontinentia pigmenti presenting with unilateral nystagmus. *Pediatr Blood Cancer*, *65*(3), e26886.

Bodak, N., Hadj-Rabia, S., Hamel-Teillac, D., de Prost, Y., & Bodemer, C. (2003). Late recurrence of inflammatory first-stage lesions in incontinentia pigmenti: an unusual phenomenon and a fascinating pathologic mechanism. *Archives of dermatology*, *139*(2), 201-204.

Bryant, S. A., & Rutledge, S. L. (2007). Abnormal white matter in a neurologically intact child with incontinentia pigmenti. *Pediatric Neurology*, *36*(3), 199-201.

Callea, M., Faletra, F., Maestro, A., Verzegnassi, F., Rabusin, M., Vinciguerra, A., Radovich, F., Clarich, G., Yavuz, I., & Tumen, E. (2011). Dental Phenotype in a Patient with Hypoidrotıc Ectodermal Dysplasia and Severe Immunodeficiency. *Journal of International Dental and Medical Research*, *4*(1), 17-20.

Carlberg, V. M., Lofgren, S. M., Mann, J. A., Austin, J. P., Nolt, D., Shereck, E. B., Davila‐Saldana, B., Zonana, J., & Krol, A. L. (2014). Hypohidrotic ectodermal dysplasia, osteopetrosis, lymphedema, and immunodeficiency in an infant with multiple opportunistic infections. *Pediatr Dermatol*, *31*(6), 716-721.

Chambelland, A., Aubert, H., Bourrat, E., Morice-Picard, F., Puzenat, E., Lacour, J., Chiaverini, C., & Group, S. F. d. D. P. R. (2020). Incontinentia pigmenti in boys: Causes and consequences. Annales de Dermatologie et de Vénéréologie,

Chang, T. T., Behshad, R., Brodell, R. T., & Gilliam, A. C. (2008). A male infant with anhidrotic ectodermal dysplasia/immunodeficiency accompanied by incontinentia pigmenti and a mutation in the NEMO pathway. *J Am Acad Dermatol*, *58*(2), 316-320.

Cheng, L. E., Kanwar, B., Tcheurekdjian, H., Grenert, J. P., Muskat, M., Heyman, M. B., McCune, J. M., & Wara, D. W. (2009). Persistent systemic inflammation and atypical enterocolitis in patients with NEMO syndrome. *Clinical Immunology*, *132*(1), 124-131.

Consortium, I. I. (2001). Survival of male patients with incontinentia pigmenti carrying a lethal mutation can be explained by somatic mosaicism or Klinefelter syndrome. *The American Journal of Human Genetics*, *69*(6), 1210-1217.

Conte, M. I., Pescatore, A., Paciolla, M., Esposito, E., Miano, M. G., Lioi, M. B., McAleer, M. A., Giardino, G., Pignata, C., & Irvine, A. D. (2014). Insight into IKBKG/NEMO locus: report of new mutations and complex genomic rearrangements leading to incontinentia pigmenti disease. *Hum Mutat*, *35*(2), 165-177.

Čulić, V., Gabrić, D., Puizina-Ivić, N., Rozman, K., Peterlin, B., & Pavelić, J. (2008). De Novo NEMO Gene Deletion (D4–10)–A Cause of Incontinentia Pigmenti in a Female Infant: A Case Report. *Collegium antropologicum*, *32*(4), 1259-1262.

Danescu, S., Has, C., Baican, C., Müller, T., & Baican, A. (2018). A novel IKBKG mutation in a patient with incontinentia pigmenti and features of hepatic ciliopathy. *Australasian Journal of Dermatology*, *59*(4), e262-e265.

Dangouloff-Ros, V., Hadj-Rabia, S., Santos, J. O., Bal, E., Desguerre, I., Kossorotoff, M., An, I., Smahi, A., Bodemer, C., & Munnich, A. (2017). Severe neuroimaging anomalies are usually associated with random X inactivation in leucocytes circulating DNA in X-linked dominant incontinentia pigmenti. *Molecular Genetics and Metabolism*, *122*(3), 140-144.

de Jesus, A. A., Hou, Y., Brooks, S., Malle, L., Biancotto, A., Huang, Y., Calvo, K. R., Marrero, B., Moir, S., & Oler, A. J. (2020). Distinct interferon signatures and cytokine patterns define additional systemic autoinflammatory diseases. *J Clin Invest*, *130*(4), 1669-1682.

Devora, G. A., Sun, L., Chen, Z., Van Oers, N. S., Hanson, E. P., Orange, J. S., & De La Morena, M. T. (2010). A novel missense mutation in the nuclear factor-κB essential modulator (NEMO) gene resulting in impaired activation of the NF-κB pathway and a unique clinical phenotype presenting as MRSA subdural empyema. *J Clin Immunol*, *30*, 881-885.

Dufke, A., Vollmer, B., Kendziorra, H., Mackensen‐Haen, S., Orth, U., Orlikowsky, T., & Gal, A. (2001). Hydrops fetalis in three male fetuses of a female with incontinentia pigmenti. *Prenatal Diagnosis: Published in Affiliation With the International Society for Prenatal Diagnosis*, *21*(12), 1019-1021.

Dupuis-Girod, S., Corradini, N., Hadj-Rabia, S., Fournet, J.-C., Faivre, L., Le Deist, F. o., Durand, P., Döffinger, R., Smahi, A., & Israel, A. (2002). Osteopetrosis, lymphedema, anhidrotic ectodermal dysplasia, and immunodeficiency in a boy and incontinentia pigmenti in his mother. *Pediatrics*, *109*(6), e97-e97.

Ergin, F. B. C., Tekin, M., Güneş, M., Güneş, B., Baysun, Ş., & Akar, N. (2022). A Turkish case of incontinentia pigmenti with a deletion mutation at Inhibitor of kappa B kinase gamma gene. *Egyptian Journal of Medical Human Genetics*, *23*(1), 6.

Faletra, F., Bruno, I., Berti, I., Pastore, S., Pirrone, A., & Tommasini, A. (2012). A red baby should not be taken too lightly. *Acta Paediatrica*, *101*(12), e573-e577.

Filipe-Santos, O., Bustamante, J., Haverkamp, M. H., Vinolo, E., Ku, C.-L., Puel, A., Frucht, D. M., Christel, K., Von Bernuth, H., & Jouanguy, E. (2006). X-linked susceptibility to mycobacteria is caused by mutations in NEMO impairing CD40-dependent IL-12 production. *The Journal of experimental medicine*, *203*(7), 1745-1759.

Franco, L. M., Goldstein, J., Prose, N. S., Selim, M. A., Tirado, C. A., Coale, M. M., & McDonald, M. T. (2006). Incontinentia pigmenti in a boy with XXY mosaicism detected by fluorescence in situ hybridization. *J Am Acad Dermatol*, *55*(1), 136-138.

Frost, M., Tencerova, M., Andreasen, C. M., Andersen, T. L., Ejersted, C., Svaneby, D., Qui, W., Kassem, M., Zarei, A., & McAlister, W. H. (2019). Absence of an osteopetrosis phenotype in IKBKG (NEMO) mutation-positive women: A case-control study. *Bone*, *121*, 243-254.

Fryssira, H., Kakourou, T., Valari, M., Stefanaki, K., Amenta, S., & Kanavakis, E. (2011). Incontinentia pigmenti revisited. A novel nonsense mutation of the IKBKG gene. *Acta Paediatrica*, *100*(1), 128-133.

Fusco, F., Bardaro, T., Fimiani, G., Mercadante, V., Miano, M. G., Falco, G., Israël, A., Courtois, G., D'Urso, M., & Ursini, M. V. (2004). Molecular analysis of the genetic defect in a large cohort of IP patients and identification of novel NEMO mutations interfering with NF-κB activation. *Human molecular genetics*, *13*(16), 1763-1773.

Fusco, F., Conte, M. I., Diociaiuti, A., Bigoni, S., Branda, M. F., Ferlini, A., El Hachem, M., & Ursini, M. V. (2017). Unusual father-to-daughter transmission of incontinentia pigmenti due to mosaicism in IP males. *Pediatrics*, *140*(3).

Fusco, F., Fimiani, G., Tadini, G., & Ursini, M. V. (2007). Clinical diagnosis of incontinentia pigmenti in a cohort of male patients. *J Am Acad Dermatol*, *56*(2), 264-267.

Gregersen, P. A., Sommerlund, M., Ramsing, M., Gjørup, H., Rasmussen, A. A., & Aggerholm, A. (2013). Diagnostic and molecular genetic challenges in male incontinentia pigmenti: a case report. *Acta Dermato-Venereologica*, *93*(6), 741-742.

Guevara, B. E. K., Hsu, C. K., Liu, L., Feast, A., Alabado, K. L. P., Lacuesta, M. P. M., Lee, J. Y. Y., & McGrath, J. A. (2016). Improved molecular diagnosis of the common recurrent intragenic deletion mutation in IKBKG in a F ilipino family with incontinentia pigmenti. *Australasian Journal of Dermatology*, *57*(2), 150-153.

Hadj-Rabia, S., Rimella, A., Smahi, A., Fraitag, S., Hamel-Teillac, D., Bonnefont, J.-P., de Prost, Y., & Bodemer, C. (2011). Clinical and histologic features of incontinentia pigmenti in adults with nuclear factor-κB essential modulator gene mutations. *J Am Acad Dermatol*, *64*(3), 508-515.

Haque, M. N., Ohtsubo, M., Nishina, S., Nakao, S., Yoshida, K., Hosono, K., Kurata, K., Ohishi, K., Fukami, M., & Sato, M. (2021). Analysis of IKBKG/NEMO gene in five Japanese cases of incontinentia pigmenti with retinopathy: fine genomic assay of a rare male case with mosaicism. *Journal of Human Genetics*, *66*(2), 205-214.

Haverkamp, M. H., Marciano, B. E., Frucht, D. M., Jain, A., van de Vosse, E., & Holland, S. M. (2014). Correlating interleukin-12 stimulated interferon-γ production and the absence of ectodermal dysplasia and anhidrosis (EDA) in patients with mutations in NF-κB essential modulator (NEMO). *J Clin Immunol*, *34*, 436-443.

Hegazy, S., Marques, M. C., Canna, S. W., Goldbach-Mansky, R., de Jesus, A. A., Reyes-Múgica, M., & Salgado, C. M. (2022). NEMO-NDAS: a panniculitis in the young representing an autoinflammatory disorder in disguise. *The American Journal of Dermatopathology*, *44*(6), e64-e66.

Heller, S., Kölsch, U., Magg, T., Krüger, R., Scheuern, A., Schneider, H., Eichinger, A., Wahn, V., Unterwalder, N., & Lorenz, M. (2020). T cell impairment is predictive for a severe clinical course in NEMO deficiency. *J Clin Immunol*, *40*, 421-434.

Hsiao, P.-F., Lin, S.-P., Chiang, S.-S., Wu, Y.-H., Chen, H.-C., & Lin, Y.-C. (2010). NEMO gene mutations in Chinese patients with incontinentia pigmenti. *Journal of the Formosan Medical Association*, *109*(3), 192-200.

Hsu, A. P., Zerbe, C. S., Foruraghi, L., Iovine, N. M., Leiding, J. W., Mushatt, D. M., Wild, L., Kuhns, D. B., & Holland, S. M. (2018). IKBKG (NEMO) 5′ untranslated splice mutations lead to severe, chronic disseminated mycobacterial infections. *Clinical Infectious Diseases*, *67*(3), 456-459.

Huang, J., Kondo, H., & Uchio, E. (2007). A case of incontinentia pigmenti in Japan and its genetic examination. *Japanese journal of ophthalmology*, *51*, 142-145.

Huang, S., Hu, C., Wang, C., & Chen, S. (2015). Incontinentia Pigmenti Associated with Seizures: A Case Report and Literature Review 色素失調症與驚厥發作的關係: 一例報導及文獻綜論. *HK J Paediatr (new series)*, *20*, 256-259.

Hubeau, M., Ngadjeua, F., Puel, A., Israel, L., Feinberg, J., Chrabieh, M., Belani, K., Bodemer, C., Fabre, I., & Plebani, A. (2011). New mechanism of X-linked anhidrotic ectodermal dysplasia with immunodeficiency: impairment of ubiquitin binding despite normal folding of NEMO protein. *Blood, The Journal of the American Society of Hematology*, *118*(4), 926-935.

Hull, S., Arno, G., Thomson, P., Mutch, S., Webster, A. R., Rai, H., Hill, V., & Moore, A. T. (2015). Somatic mosaicism of a novel IKBKG mutation in a male patient with incontinentia pigmenti. *American Journal of Medical Genetics Part A*, *167*(7), 1601-1604.

Huppmann, A. R., Leiding, J. W., Hsu, A. P., Raffeld, M., Uzel, G., Pittaluga, S., & Holland, S. M. (2015). Pathologic findings in NEMO deficiency: a surgical and autopsy survey. *Pediatric and Developmental Pathology*, *18*(5), 387-400.

Huttner, H., Richter, G., Jünemann, A., Kress, W., Weis, J., Schröder, J., Gal, A., Doerfler, A., Udd, B., & Schröder, R. (2010). Incontinetia pigmenti-related myopathy or unsolved “double trouble”? *Neuromuscular Disorders*, *20*(2), 139-141.

Imamura, M., Kawai, T., Okada, S., Izawa, K., Takachi, T., Iwabuchi, H., Yoshida, S., Hosokai, R., Kanegane, H., & Yamamoto, T. (2011). Disseminated BCG infection mimicking metastatic nasopharyngeal carcinoma in an immunodeficient child with a novel hypomorphic NEMO mutation. *J Clin Immunol*, *31*, 802-810.

Inaba, S., Aizawa, Y., Miwa, Y., Imai, C., Ohnishi, H., Kanegane, H., & Saitoh, A. (2021). Case report: Analysis of preserved umbilical cord clarified X-linked anhidrotic ectodermal dysplasia with immunodeficiency in deceased, undiagnosed uncles. *Frontiers in immunology*, *12*, 786164.

Inoue, Y., Shimizu, A., Suto, M., Kishi, C., Takahashi, A., Yasuda, M., Iijima, M., Arakawa, H., & Ishikawa, O. (2018). Cutaneous squamous cell carcinoma, thyroid cancer and Langerhans cell histiocytosis in a patient with X‐linked recessive Mendelian susceptibility to mycobacterial diseases with a nuclear factor‐κB essential modifier mutation. *J Dermatol*, *45*(8), 1017-1019.

Jain, A., Ma, C. A., Liu, S., Brown, M., Cohen, J., & Strober, W. (2001). Specific missense mutations in NEMO result in hyper-IgM syndrome with hypohydrotic ectodermal dysplasia. *Nat Immunol*, *2*(3), 223-228.

Jiang, J., Zeng, J., He, Q., Yang, J., Wang, S., & Zhang, Z. (2022). NEMO gene mutations in two Chinese females with incontinentia pigmenti. *Clinical, Cosmetic and Investigational Dermatology*, 815-821.

Johnston, A. M., Niemela, J., Rosenzweig, S. D., Fried, A. J., Delmonte, O. M., Fleisher, T. A., & Kuehn, H. (2016). A novel mutation in IKBKG/NEMO leads to ectodermal dysplasia with severe immunodeficiency (EDA-ID). *J Clin Immunol*, *36*, 541-543.

Kanai, S., Okanishi, T., Kawai, M., Yoshino, G., Tsubouchi, Y., Nishimura, Y., Sakuma, H., Kurahashi, H., & Maegaki, Y. (2021). Late-onset cerebral arteriopathy in a patient with incontinentia pigmenti. *Brain and Development*, *43*(4), 580-584.

Karakawa, S., Okada, S., Tsumura, M., Mizoguchi, Y., Ohno, N., Yasunaga, S. i., Ohtsubo, M., Kawai, T., Nishikomori, R., & Sakaguchi, T. (2011). Decreased expression in nuclear factor-κB essential modulator due to a novel splice-site mutation causes X-linked ectodermal dysplasia with immunodeficiency. *J Clin Immunol*, *31*, 762-772.

Karamchandani-Patel, G., Hanson, E. P., Saltzman, R., Kimball, C. E., Sorensen, R. U., & Orange, J. S. (2011). Congenital alterations of glutamic acid 223 in the second NEMO alpha-helix result in Hypohidrotic Ectodermal Dysplasia and Immunodeficiency with Normal Serum IgG Levels. *Annals of allergy, asthma & immunology: official publication of the American College of Allergy, Asthma, & Immunology*, *107*(1), 50.

Kawai, M., Sugimoto, A., Ishihara, Y., Kato, T., & Kurahashi, H. (2022). Incontinentia pigmenti inherited from a father with a low level atypical IKBKG deletion mosaicism: a case report. *BMC pediatrics*, *22*(1), 378.

Kawai, T., Nishikomori, R., Izawa, K., Murata, Y., Tanaka, N., Sakai, H., Saito, M., Yasumi, T., Takaoka, Y., & Nakahata, T. (2012). Frequent somatic mosaicism of NEMO in T cells of patients with X-linked anhidrotic ectodermal dysplasia with immunodeficiency. *Blood, The Journal of the American Society of Hematology*, *119*(23), 5458-5466.

Keller, M. D., Petersen, M., Ong, P., Risma, K., Burnham, J., Stiehm, E., Hanson, E. P., Deardorff, M. A., & Orange, J. S. (2011). Hypohidrotic ectodermal dysplasia and immunodeficiency with coincident NEMO and EDA mutations. *Frontiers in immunology*, *2*, 16178.

Khan, T. A., Schimke, L. F., Amaral, E. P., Ishfaq, M., Barbosa Bonfim, C. C., Rahman, H., Iqbal, A., D'Imperio Lima, M. R., Costa Carvalho, B. T., & Cabral‐Marques, O. (2016). Interferon‐gamma reduces the proliferation of M. tuberculosis within macrophages from a patient with a novel hypomorphic NEMO mutation. *Pediatr Blood Cancer*, *63*(10), 1863-1866.

Kibbi, N., Totonchy, M., Suozzi, K. C., Ko, C. J., & Odell, I. D. (2018). A case of subungual tumors of incontinentia pigmenti: A rare manifestation and association with bipolar disease. *JAAD Case Rep*, *4*(7), 737-741.

Kim, H. Y., Song, H. B., Kim, K. H., Kim, J. H., Chae, J. H., Kim, M. J., Seong, M. W., & Ko, J. M. (2021). Importance of extracutaneous organ involvement in determining the clinical severity and prognosis of incontinentia pigmenti caused by mutations in the IKBKG gene. *Experimental Dermatology*, *30*(5), 676-683.

Kim, M. J., Lyu, S. W., Seok, H. H., Park, J. E., Shim, S. H., & Yoon, T. K. (2014). A healthy delivery of twins by assisted reproduction followed by preimplantation genetic screening in a woman with X-linked dominant incontinentia pigmenti. *Clinical and Experimental Reproductive Medicine*, *41*(4), 168.

Kiritsi, D., Valari, M., Michos, A., Karakosta, V., & Has, C. (2016). The mysteries of mosaicism: phenotypic variability in a family with incontinentia pigmenti. *European Journal of Dermatology*, *26*(5), 504-506.

Klemann, C., Pannicke, U., Morris-Rosendahl, D. J., Vlantis, K., Rizzi, M., Uhlig, H., Vraetz, T., Speckmann, C., Strahm, B., & Pasparakis, M. (2016). Transplantation from a symptomatic carrier sister restores host defenses but does not prevent colitis in NEMO deficiency. *Clinical Immunology*, *164*, 52-56.

Kmetz, E. C., Shashidhar Pai, G., & Burges, G. E. (2009). Incontinentia Pigmenti with a Foreshortened Hand: Evidence for the Significance of NFκB in Human Morphogenesis. *Pediatr Dermatol*, *26*(1), 83-86.

Kolitz, E., Chamseddin, B., Son, R., Vandergriff, T., Hsu, A. P., Holland, S., & Wang, R. C. (2021). A novel NEMO/IKBKG mutation identified in a primary immunodeficiency disorder with recurrent atypical mycobacterial infections. *JAAD Case Rep*, *7*, 33-35.

Ku, C.-L., Dupuis-Girod, S., Dittrich, A.-M., Bustamante, J., Santos, O. F., Schulze, I., Bertrand, Y., Couly, G., Bodemer, C., & Bossuyt, X. (2005). NEMO mutations in 2 unrelated boys with severe infections and conical teeth. *Pediatrics*, *115*(5), e615-e619.

Ku, C.-L., Picard, C., Erdös, M., Jeurissen, A., Bustamante, J., Puel, A., von Bernuth, H., Filipe-Santos, O., Chang, H.-H., & Lawrence, T. (2007). IRAK4 and NEMO mutations in otherwise healthy children with recurrent invasive pneumococcal disease. *Journal of medical genetics*, *44*(1), 16-23.

Lee, Y., Kim, S., Kim, K., & Chang, M. (2011). Incontinentia pigmenti in a newborn with NEMO mutation. *Journal of Korean medical science*, *26*(2), 308.

Lee, Y., Wessel, A. W., Xu, J., Reinke, J. G., Lee, E., Kim, S. M., Hsu, A. P., Zilberman-Rudenko, J., Cao, S., & Enos, C. (2022). Genetically programmed alternative splicing of NEMO mediates an autoinflammatory disease phenotype. *J Clin Invest*, *132*(6).

Liao, S.-L., Lai, S.-H., Huang, J.-L., Lee, N.-C., & Lee, W.-I. (2013). Serial cytokine expressions in infants with incontinentia pigmenti. *Immunobiology*, *218*(5), 772-779.

Loh, N. R., Jadresic, L. P., & Whitelaw, A. (2008). A genetic cause for neonatal encephalopathy: incontinentia pigmenti with NEMO mutation. *Acta Paediatrica*, *97*(3), 379-381.

Maingay-de Groof, F., Lequin, M. H., Roofthooft, D. W., Oranje, A. P., de Coo, I. F., Bok, L. A., van der Spek, P. J., Mancini, G. M., & Govaert, P. P. (2008). Extensive cerebral infarction in the newborn due to incontinentia pigmenti. *European Journal of Paediatric Neurology*, *12*(4), 284-289.

Mancini, A. J., Lawley, L. P., & Uzel, G. (2008). X-linked ectodermal dysplasia with immunodeficiency caused by NEMO mutation: early recognition and diagnosis. *Archives of dermatology*, *144*(3), 342-346.

Mansour, S., Woffendin, H., Mitton, S., Jeffery, I., Jakins, T., Kenwrick, S., & Murday, V. (2001). Incontinentia pigmenti in a surviving male is accompanied by hypohidrotic ectodermal dysplasia and recurrent infection. *Am J Med Genet*, *99*(2), 172-177.

Margari, L., Lamanna, A. L., Buttiglione, M., Craig, F., Petruzzelli, M. G., & Terenzio, V. (2013). Long-term follow-up of neurological manifestations in a boy with incontinentia pigmenti. *Eur J Pediatr*, *172*, 1259-1262.

Mariath, L. M., Santa Maria, F. D., Poziomczyk, C. S., Travi, G. M., Wachholz, G. E., De Souza, S. R., Kiszewski, A. E., & Schuler‐Faccini, L. (2018). Intrafamilial clinical variability in four families with incontinentia pigmenti. *American Journal of Medical Genetics Part A*, *176*(11), 2318-2324.

Martinez-Pomar, N., Munoz-Saa, I., Heine-Suner, D., Martin, A., Smahi, A., & Matamoros, N. (2005). A new mutation in exon 7 of NEMO gene: late skewed X-chromosome inactivation in an incontinentia pigmenti female patient with immunodeficiency. *Human genetics*, *118*, 458-465.

Martuszewski, A., Paluszkiewicz, P., Sierżęga-Staykov, K., Wawrzyniak-Dzierżek, E., Salamonowicz-Bodzioch, M., Frączkiewicz, J., Janeczko-Czarnecka, M., Mielcarek-Siedziuk, M., Nowak, M., & Dąbrowska-Leonik, N. (2020). Successful Allogeneic Stem Cell Transplantation in Nuclear Factor-Kappa B Essential Modulator Deficiency Syndrome After Treosulfan-Based Conditioning: A Case Report. Transplantation Proceedings,

Matsumoto, N., Takahashi, S., Toriumi, N., Sarashina, T., Makita, Y., Tachibana, Y., & Fujieda, K. (2009). Acute disseminated encephalomyelitis in an infant with incontinentia pigmenti. *Brain and Development*, *31*(8), 625-628.

Matsuzaki, Y., Rokunohe, A., Minakawa, S., Nomura, K., Nakano, H., Ito, E., & Sawamura, D. (2018). Incontinentia pigmenti in a male (XY) infant with long‐term follow up over 8 years. *J Dermatol*, *45*(1), 100-103.

Mayer, E., Shuttleworth, G., Greenhalgh, K., Sansom, J., Grey, R., & Kenwrick, S. (2003). Novel corneal features in two males with incontinentia pigmenti. *British journal of ophthalmology*, *87*(5), 554-556.

Minakawa, S., Takeda, H., Nakano, H., Tono, C., Takahashi, Y., Sasaki, S., Terui, K., Ito, E., & Sawamura, D. (2009). Successful umbilical cord blood transplantation for intractable eczematous eruption in hypohidrotic ectodermal dysplasia with immunodeficiency. *Clinical and experimental dermatology*, *34*(7), e441-e442.

Minić, S., Trpinac, D., Gabriel, H., Gencik, M., & Obradović, M. (2013). First IKBKG gene mutation study in Serbian incontinentia pigmenti patients. *Srpski arhiv za celokupno lekarstvo*, *141*(7-8), 490-494.

Minić, S., Trpinac, D., Novaković, I., Cerovac, N., Dobrosavljević Vukojević, D., & Rosain, J. (2022). Challenges in rare diseases diagnostics: Incontinentia pigmenti with heterozygous GBA mutation. *Diagnostics*, *12*(7), 1711.

Minić, S., Trpinac, D., & Obradović, M. (2015). A novel frameshift mutation of the IKBKG gene causing typical incontinentia pigmenti. *Srpski arhiv za celokupno lekarstvo*, *143*(11-12), 752-754.

Mizukami, T., Obara, M., Nishikomori, R., Kawai, T., Tahara, Y., Sameshima, N., Marutsuka, K., Nakase, H., Kimura, N., & Heike, T. (2012). Successful treatment with infliximab for inflammatory colitis in a patient with X-linked anhidrotic ectodermal dysplasia with immunodeficiency. *J Clin Immunol*, *32*, 39-49.

Mizuno, M., Aso, K., Tsuzuki, Y., Kitazawa, T., Migita, O., Hokuto, I., & Yamamoto, H. (2020). A successful treatment of tadalafil in incontinentia pigmenti with pulmonary hypertension. *European Journal of Medical Genetics*, *63*(3), 103764.

Moosajee, M., Ali, M. A., & Wong, S. C. (2018). Retinal angiography findings in male infant with incontinentia pigmenti and sickle cell trait. *JAMA ophthalmology*, *136*(11), e183140-e183140.

Moro, R., Fabiano, A., Calzavara-Pinton, P., Cardinale, J., Palumbo, G., Giliani, S., Lanzi, G., Antonelli, F., De Simone, M., & Martelli, P. (2020). Incontinentia Pigmenti Associated with Aplasia Cutis Congenita in a Newborn Male with Klinefelter Syndrome: Is the Severity of Neurological Involvement Linked to Skin Manifestations? *Dermatology and Therapy*, *10*, 213-220.

Mullan, E., Barbarian, M., Trakadis, Y., & Moroz, B. (2014). Incontinentia pigmenti in an XY boy: case report and review of the literature. *Journal of Cutaneous Medicine and Surgery*, *18*(2), 119-122.

Nicolaou, N., & Graham‐Brown, R. (2003). Nail dystrophy, an unusual presentation of incontinentia pigmenti. *British Journal of Dermatology*, *149*(6), 1286-1288.

Niehues, T., Reichenbach, J., Neubert, J., Gudowius, S., Puel, A., Horneff, G., Lainka, E., Dirksen, U., Schroten, H., & Döffinger, R. (2004). Nuclear factor κB essential modulator–deficient child with immunodeficiency yet without anhidrotic ectodermal dysplasia. *Journal of allergy and clinical immunology*, *114*(6), 1456-1462.

Nishikomori, R., Akutagawa, H., Maruyama, K., Nakata-Hizume, M., Ohmori, K., Mizuno, K., Yachie, A., Yasumi, T., Kusunoki, T., & Heike, T. (2004). X-linked ectodermal dysplasia and immunodeficiency caused by reversion mosaicism of NEMO reveals a critical role for NEMO in human T-cell development and/or survival. *Blood*, *103*(12), 4565-4572.

Ogasawara, K., Honda, Y., Maeda, H., Sato, M., Nakano, H., & Hosoya, M. (2019). Corticosteroid therapy in neonatal incontinentia pigmenti with asymptomatic cerebral lesions. *Pediatric Neurology*, *99*, 85-87.

Ohnishi, H., Kishimoto, Y., Taguchi, T., Kawamoto, N., Nakama, M., Kawai, T., Nakayama, M., Ohara, O., Orii, K., & Fukao, T. (2017). Immunodeficiency in two female patients with incontinentia pigmenti with heterozygous NEMO mutation diagnosed by LPS unresponsiveness. *J Clin Immunol*, *37*, 529-538.

Okita, M., Nakanishi, G., Fujimoto, N., Shiomi, M., Yamada, T., Wataya‐Kaneda, M., Takijiri, C., Yokoyama, Y., Sunohara, A., & Tanaka, T. (2013). NEMO gene rearrangement (exon 4–10 deletion) and genotype–phenotype relationship in J apanese patients with incontinentia pigmenti and review of published work in J apanese patients. *J Dermatol*, *40*(4), 272-276.

Onnis, G., Diociaiuti, A., Zangari, P., D'Argenio, P., Cancrini, C., Iughetti, L., & El Hachem, M. (2018). Cardiopulmonary anomalies in incontinentia pigmenti patients. *International journal of dermatology*, *57*(1), 40-45.

Orange, J. S., Brodeur, S. R., Jain, A., Bonilla, F. A., Schneider, L. C., Kretschmer, R., Nurko, S., Rasmussen, W. L., Köhler, J. R., & Gellis, S. E. (2002). Deficient natural killer cell cytotoxicity in patients with IKK-γ/NEMO mutations. *J Clin Invest*, *109*(11), 1501-1509.

Orange, J. S., Jain, A., Ballas, Z. K., Schneider, L. C., Geha, R. S., & Bonilla, F. A. (2004). The presentation and natural history of immunodeficiency caused by nuclear factor κB essential modulator mutation. *Journal of allergy and clinical immunology*, *113*(4), 725-733.

Orange, J. S., Levy, O., Brodeur, S. R., Krzewski, K., Roy, R. M., Niemela, J. E., Fleisher, T. A., Bonilla, F. A., & Geha, R. S. (2004). Human nuclear factor κB essential modulator mutation can result in immunodeficiency without ectodermal dysplasia. *Journal of allergy and clinical immunology*, *114*(3), 650-656.

Ørstavik, K. H., Kristiansen, M., Knudsen, G. P., Storhaug, K., Vege, Å., Eiklid, K., Abrahamsen, T. G., Smahi, A., & Steen‐Johnsen, J. (2006). Novel splicing mutation in the NEMO (IKK‐gamma) gene with severe immunodeficiency and heterogeneity of X‐chromosome inactivation. *American Journal of Medical Genetics Part A*, *140*(1), 31-39.

Pai, S.-Y., Levy, O., Jabara, H. H., Glickman, J. N., Stoler-Barak, L., Sachs, J., Nurko, S., Orange, J. S., & Geha, R. S. (2008). Allogeneic transplantation successfully corrects immune defects, but not susceptibility to colitis, in a patient with nuclear factor-κB essential modulator deficiency. *Journal of allergy and clinical immunology*, *122*(6), 1113-1118. e1111.

Pauly, E., Linderkamp, O., & Pöschl, J. (2005). Incontinentia pigmenti in combination with decreased IgG subclass concentrations in a female newborn. *Neonatology*, *88*(3), 172-174.

Pengelly, R., Upstill‐Goddard, R., Arias, L., Martinez, J., Gibson, J., Knut, M., Collins, A. L., Ennis, S., Collins, A., & Briceno, I. (2015). Resolving clinical diagnoses for syndromic cleft lip and/or palate phenotypes using whole‐exome sequencing. *Clinical genetics*, *88*(5), 441-449.

Permaul, P., Narla, A., Hornick, J. L., & Pai, S.-Y. (2009). Allogeneic hematopoietic stem cell transplantation for X-linked ectodermal dysplasia and immunodeficiency: case report and review of outcomes. *Immunol Res*, *44*, 89-98.

Phan, T., Wargon, O., & Turner, A. (2005). Incontinentia pigmenti case series: clinical spectrum of incontinentia pigmenti in 53 female patients and their relatives. *Clinical and experimental dermatology*, *30*(5), 474-480.

Piccoli, G. B., Attini, R., Vigotti, F., Naretto, C., Fassio, F., Randone, O., Restagno, G., Todros, T., & Roccatello, D. (2012). NEMO syndrome (incontinentia pigmenti) and systemic lupus erythematosus: a new disease association. *Lupus*, *21*(6), 675-681.

Pizzamiglio, M. R., Piccardi, L., Bianchini, F., Canzano, L., Palermo, L., Fusco, F., D'Antuono, G., Gelmini, C., Garavelli, L., & Ursini, M. V. (2014). Incontinentia pigmenti: learning disabilities are a fundamental hallmark of the disease. *PLoS One*, *9*(1), e87771.

Rae, W., Ward, D., Mattocks, C. J., Gao, Y., Pengelly, R. J., Patel, S. V., Ennis, S., Faust, S. N., & Williams, A. P. (2017). Autoimmunity/inflammation in a monogenic primary immunodeficiency cohort. *Clinical & translational immunology*, *6*(9), e155.

Ramírez-Alejo, N., Alcántara-Montiel, J. C., Yamazaki-Nakashimada, M., Duran-McKinster, C., Valenzuela-León, P., Rivas-Larrauri, F., Cedillo-Barrón, L., Hernández-Rivas, R., & Santos-Argumedo, L. (2015). Novel hypomorphic mutation in IKBKG impairs NEMO-ubiquitylation causing ectodermal dysplasia, immunodeficiency, incontinentia pigmenti, and immune thrombocytopenic purpura. *Clinical Immunology*, *160*(2), 163-171.

Rashidghamat, E., Hsu, C.-K., Nanda, A., Liu, L., Al‐Ajmi, H., & McGrath, J. A. (2016). Incontinentia pigmenti in a father and daughter. *British Journal of Dermatology*, *175*(5), 1059-1060.

Rheault, S. (2021). Severe COVID-19 and long COVID in a 31-year-old woman with incontinentia pigmenti: A case report. *SAGE Open Medical Case Reports*, *9*, 2050313X211059295.

Ricci, S., Romano, F., Nieddu, F., Picard, C., & Azzari, C. (2017). OL-EDA-ID syndrome: a novel hypomorphic NEMO mutation associated with a severe clinical presentation and transient HLH. *J Clin Immunol*, *37*, 7-11.

Roberts, C. M., Angus, J. E., Leach, I. H., McDermott, E. M., Walker, D. A., & Ravenscroft, J. C. (2010). A novel NEMO gene mutation causing osteopetrosis, lymphoedema, hypohidrotic ectodermal dysplasia and immunodeficiency (OL-HED-ID). *Eur J Pediatr*, *169*, 1403-1407.

Salt, B. H., Niemela, J. E., Pandey, R., Hanson, E. P., Deering, R. P., Quinones, R., Jain, A., Orange, J. S., & Gelfand, E. W. (2008). IKBKG (nuclear factor-κB essential modulator) mutation can be associated with opportunistic infection without impairing Toll-like receptor function. *Journal of allergy and clinical immunology*, *121*(4), 976-982.

Sanka, R., & Kumar, M. (2004). An unusual newborn rash. *Fetal and Pediatric Pathology*, *23*(4), 275-279.

Schmid, J. M. P., Junge, S. A., Hossle, J. P., Schneider, E. M., Roosnek, E., Seger, R. A., & Gungor, T. (2006). Transient hemophagocytosis with deficient cellular cytotoxicity, monoclonal immunoglobulin M gammopathy, increased T-cell numbers, and hypomorphic NEMO mutation. *Pediatrics*, *117*(5), e1049-e1056.

Seo, M. Y., You, S. J., Kim, S. H., Cho, W. H., & Chae, J. H. (2017). A 6-month-old girl with Incontinentia Pigmenti presenting as status epilepticus. *Journal of Epilepsy Research*, *7*(2), 118.

Silan, F., Aydogan, I., Kavak, A., Bardaro, T., & D’Urso, M. (2004). Incontinentia pigmenti with NEMO mutation in a Turkish family. *International journal of dermatology*, *43*(7), 527-529.

Soltirovska Salamon, A., Lichtenbelt, K., Cowan, F. M., Casaer, A., Dudink, J., Dereymaeker, A., Paro‐Panjan, D., Groenendaal, F., & de Vries, L. S. (2016). Clinical presentation and spectrum of neuroimaging findings in newborn infants with incontinentia pigmenti. *Developmental Medicine & Child Neurology*, *58*(10), 1076-1084.

Song, M.-J., Chae, J.-H., Park, E.-A., & Ki, C.-S. (2010). The common NF-κB essential modulator (NEMO) gene rearrangement in Korean patients with incontinentia pigmenti. *Journal of Korean medical science*, *25*(10), 1513.

Su, P.-H., Chen, J.-Y., Yu, J.-S., Su, C.-M., Huang, T.-C., & Chen, S.-J. (2004). De Novo incontinentia pigmenti in female twins. *Acta Paediatrica Taiwanica= Taiwan er ke yi xue hui za zhi*, *45*(3), 178-180.

Sun, S., Li, F., Liu, Y., Qu, H., Wong, S.-W., Zeng, L., Yu, M., Feng, H., Liu, H., & Han, D. (2019). A novel inhibitor of nuclear factor kappa-B kinase subunit gamma mutation identified in an incontinentia pigmenti patient with syndromic tooth agenesis. *Arch Oral Biol*, *101*, 100-107.

Surucu Yilmaz, N., Bilgic Eltan, S., Kayaoglu, B., Geckin, B., Heredia, R. J., Sefer, A. P., Kiykim, A., Nain, E., Kasap, N., & Dogru, O. (2022). Low density granulocytes and dysregulated neutrophils driving autoinflammatory manifestations in NEMO deficiency. *J Clin Immunol*, 1-15.

Takada, H., Nomura, A., Ishimura, M., Ichiyama, M., Ohga, S., & Hara, T. (2010). NEMO mutation as a cause of familial occurrence of Behçet's disease in female patients. *Clinical genetics*, *78*(6), 575-579.

Thakur, S., Puri, R. D., Kohli, S., Saxena, R., & Verma, I. (2011). Utility of molecular studies in incontinentia pigmenti patients. *Indian Journal of Medical Research*, *133*(4), 442-445.

Tono, C., Takahashi, Y., Terui, K., Sasaki, S., Kamio, T., Tandai, S., Sato, T., Kudo, K., Toki, T., & Tachibana, N. (2007). Correction of immunodeficiency associated with NEMO mutation by umbilical cord blood transplantation using a reduced-intensity conditioning regimen. *Bone marrow transplantation*, *39*(12), 801-804.

Toyohara, M., Kajiho, Y., Toyofuku, E., Takahashi, C., Owada, K., Kanda, S., Harita, Y., Ohnishi, H., Wada, T., & Imai, K. (2021). An infant with X‐linked anhidrotic ectodermal dysplasia with immunodeficiency presenting with Pneumocystis pneumonia: A case report. *Clinical Case Reports*, *9*(11), e05093.

Türkmen, M., Eliaçik, K., Temoçin, K., Savk, E., Tosun, A., & Dikicioğlu, E. (2007). A rare cause of neonatal seizure: incontinentia pigmenti. *The Turkish Journal of Pediatrics*, *49*(3), 327-330.

Veronese, F., Zavattaro, E., Monzani, A., Zaffaroni, M., Landucci, G., De Miglio, L., Nugnes, M., & Savoia, P. (2018). A female newborn with papulovesicular lesions. *JDDG: Journal der Deutschen Dermatologischen Gesellschaft*, *16*(11), 1383-1386.

Wang, R., Lara-Corrales, I., Kannu, P., & Pope, E. (2019). Unraveling incontinentia pigmenti: a comparison of phenotype and genotype variants. *J Am Acad Dermatol*, *81*(5), 1142-1149.

Wang, Y., Chen, Y., Wang, Q., Wang, G., Guo, C., Wang, F., Deng, X., & Wang, W. (2013). A 14-year-old girl with an unusual combination of incontinentia pigmenti and conversion disorder. *International Journal of Clinical and Experimental Medicine*, *6*(10), 1006.

Williams, A., Chandrashekar, L., Srivastava, V. M., Thomas, M., Horo, S., & George, R. (2017). Incontinentia pigmenti, an x-linked dominant disorder, in a 2-year-old boy with Klinefelter syndrome. *Indian Journal of Pathology and Microbiology*, *60*(3), 424-426.

Wolf, D. S., Golden, W. C., Hoover-Fong, J., Applegate, C., Cohen, B. A., Germain-Lee, E. L., Goldberg, M. F., Crawford, T. O., & Gauda, E. B. (2015). High-dose glucocorticoid therapy in the management of seizures in neonatal incontinentia pigmenti: a case report. *Journal of child neurology*, *30*(1), 100-106.

Zafeiriou, D. I., Vargiami, E., Hatzidimitriou, V., & Kyriazi, M. (2013). Incontinentia pigmenti: a skin, brain, and eye matter. *The Journal of Pediatrics*, *163*(5), 1520.

Zhang, R. Z., Zhu, W. Y., & Zhou, L. (2014). Unusual hyperpigmented patches, an undeveloped breast and a cataract in a female with incontinentia pigmenti. *J Dermatol*, *41*(4), 325-328.

Zonana, J., Elder, M. E., Schneider, L. C., Orlow, S. J., Moss, C., Golabi, M., Shapira, S. K., Farndon, P. A., Wara, D. W., & Emmal, S. A. (2000). A novel X-linked disorder of immune deficiency and hypohidrotic ectodermal dysplasia is allelic to incontinentia pigmenti and due to mutations in IKK-gamma (NEMO). *The American Journal of Human Genetics*, *67*(6), 1555-1562.

Zou, C. C., & Zhao, Z. Y. (2007). Clinical and molecular analysis of NF‐κB essential modulator in Chinese incontinentia pigmenti patients. *International journal of dermatology*, *46*(10), 1017-1022.
