# Supplementary material for: Clinical relevance of loss-of-function mutations of NEMO/IKBKG
Source: Genes Dis. 2025 Jan 12;12(5):101531. doi: 10.1016/j.gendis.2025.101531 (PMC12221755; doi:10.1016/j.gendis.2025.101531)
Supplement: Multimedia component 2 [file mmc2.docx]

**Supplementary Table 1A.** Quality and risk assessment of case reports.

| **(Author and Date) Studies** | **DOI / PMID / Download Link** | **Dose the patient(s) represent(s) the whole experience of the investigator(centre) or is the selection method unclear to the extent that other patients with similar presentation may not have been reported?**  **(Y-Yes, N-No)** | **Was the exposure (NEMO mutation) adequately ascertained?**  **(Y-Yes, N-No)** | **Was the outcome adequately ascertained?**  **(Y-Yes, N-No)** | **Was follow-up long enough for outcomes to occur?**  **(Y-Yes, N-No)** | **Is the case(s) described with sufficient details to allow other investigators to replicate the research or to allow practitioners make inferences related to their own practice?**  **(Y-Yes, N-No)** | **Risk of bais**  **(＜3 high risk,**  **=3 middle risk,**  **＞3 low risk)** |
| --- | --- | --- | --- | --- | --- | --- | --- |
| (Kim et al., 2021) Importance of extracutaneous organ involvement in determining the clinical severity and prognosis of incontinentia pigmenti caused by mutations in the IKBKG gene | 10.1111/exd.14313 | Y | Y | Y | N | Y | 4 |
| (Chambelland et al., 2020) Incontinentia pigmenti in boys: Causes and consequences | 10.1016/j.annder.2019.07.007 | Y | Y | Y | N | Y | 4 |
| (Wang et al., 2019) Unraveling incontinentia pigmenti: A comparison of phenotype and genotype variants | 10.1016/j.jaad.2019.01.093 | Y | Y | Y | N | Y | 4 |
| (Mariath et al., 2018) Intrafamilial clinical variability in four families with incontinentia pigmenti | 10.1002/ajmg.a.40497 | Y | Y | Y | N | Y | 4 |
| (Dangouloff-Ros et al., 2017) Severe neuroimaging anomalies are usually associated with random X inactivation in leucocytes circulating DNA in X-linked dominant Incontinentia Pigmenti | 10.1016/j.ymgme.2017.07.001 | Y | Y | Y | N | Y | 4 |
| (Soltirovska Salamon et al., 2016) Clinical presentation and spectrum of neuroimaging findings in newborn infants with incontinentia pigmenti | 10.1111/dmcn.13140 | Y | N | Y | Y | Y | 4 |
| (Haverkamp et al., 2014) Correlating Interleukin-12 Stimulated Interferon-gamma Production and the Absence of Ectodermal Dysplasia and Anhidrosis (EDA) in Patients with Mutations in NF-kappa B Essential Modulator (NEMO) | 10.1007/s10875-014-9998-2 | N | Y | Y | Y | Y | 4 |
| (Conte et al., 2014) Insight into IKBKG/NEMO Locus: Report of New Mutations and Complex Genomic Rearrangements Leading to Incontinentia Pigmenti Disease | 10.1002/humu.22483 | Y | Y | Y | N | Y | 4 |
| (Liao et al., 2013) Serial cytokine expressions in infants with incontinentia pigmenti | 10.1016/j.imbio.2012.08.280 | Y | Y | Y | N | Y | 4 |
| (Okita et al., 2013) NEMO gene rearrangement (exon 4-10 deletion) and genotypephenotype relationship in Japanese patients with incontinentia pigmenti and review of published work in Japanese patients | 10.1111/1346-8138.12091 | Y | N | Y | N | N | 2 |
| (Faletra et al., 2012) A red baby should not be taken too lightly | 10.1111/apa.12018 | Y | Y | Y | N | Y | 4 |
| (Kawai et al., 2012) Frequent somatic mosaicism of NEMO in T cells of patients with X-linked anhidrotic ectodermal dysplasia with immunodeficiency | 10.1182/blood-2011-05-354167 | Y | Y | Y | N | N | 3 |
| (Hadj-Rabia et al., 2011) Clinical and histologic features of incontinentia pigmenti in adults with nuclear factor-κB essential modulator gene mutations | 10.1016/j.jaad.2010.01.045 | Y | N | Y | N | Y | 3 |
| (Fryssira et al., 2011) Incontinentia pigmenti revisited. A novel nonsense mutation of the IKBKG gene | 10.1111/j.1651-2227.2010.01921.x | Y | Y | Y | Y | Y | 5 |
| (Hsiao et al., 2010) NEMO Gene Mutations in Chinese Patients With Incontinentia Pigmenti | 10.1016/S0929-6646(10)60042-3 | Y | Y | Y | N | Y | 4 |
| (Cheng et al., 2009) Persistent systemic inflammation and atypical enterocolitis in patients with NEMO syndrome | 10.1016/j.clim.2009.03.514 | Y | Y | Y | N | Y | 4 |
| (Zou & Zhao, 2007) Clinical and molecular analysis of NF-κB essential modulator in Chinese incontinentia pigmenti patients | 10.1111/j.1365-4632.2007.03365.x | Y | Y | Y | N | Y | 4 |
| (Ardelean & Pope, 2006) Incontinentia pigmenti in boys: A series and review of the literature | 10.1111/j.1525-1470.2006.00302.x | Y | N | Y | N | Y | 3 |
| (Phan et al., 2005) Incontinentia pigmenti case series: clinical spectrum of incontinentia pigmenti in 53 female patients and their relatives | 10.1111/j.1365-2230.2005.01848.x | Y | Y | Y | N | Y | 4 |
| (Orange, Jain, et al., 2004) The presentation and natural history of immunodeficiency caused by nuclear factor κB essential modulator mutation | 10.1016/j.jaci.2004.01.762 | Y | Y | Y | Y | Y | 5 |
| (Bodak et al., 2003) Late recurrence of inflammatory first-stage lesions in incontinentia pigmenti: An unusual phenomenon and a fascinating pathologic mechanism | 10.1001/archderm.139.2.201 | Y | Y | Y | N | Y | 4 |
| (Consortium, 2001) Survival of male patients with incontinentia pigmenti carrying a lethal mutation can be explained by somatic mosaicism or Klinefelter syndrome | 10.1086/324591 | Y | Y | Y | Y | Y | 5 |
| (Zonana et al., 2000) A novel X-linked disorder of immune deficiency and hypohidrotic ectodermal dysplasia is allelic to incontinentia pigmenti and due to mutations in IKK-gamma (NEMO) | 10.1086/316914 | Y | Y | Y | Y | Y | 5 |
| (Thakur et al., 2011) Utility of molecular studies in incontinentia pigmenti patients | PMID: 21537100 | Y | Y | Y | N | Y | 4 |
| (Huppmann et al., 2015) Pathologic findings in NEMO deficiency: A surgical and autopsy survey | 10.2350/15-05-1631-OA.1 | N | Y | Y | N | Y | 3 |
| (Abbott et al., 2014) Successful hematopoietic cell transplantation in patients with unique NF-κB essential modulator (NEMO) mutations | 10.1038/bmt.2014.157 | Y | Y | Y | Y | N | 4 |
| (Alshenqiti et al., 2017) Pulmonary hypertension and vasculopathy in incontinentia pigmenti: A case report | 10.2147/TCRM.S134705 | Y | Y | Y | Y | Y | 5 |
| (Alkan et al., 2021) Management of COVID-19 pneumonia in a child with NEMO deficiency | 10.1007/s12026-021-09184-6 | Y | Y | Y | Y | Y | 5 |
| (Aradhya et al., 2001) Atypical forms of incontinentia pigmenti in male individuals result from mutations of a cytosine tract in exon 10 of NEMO (IKK-gamma) | 10.1086/318806 | Y | Y | Y | N | Y | 4 |
| (Artac et al., 2019) Infliximab therapy for inflammatory colitis in an infant with NEMO deficiency | 10.1007/s12026-019-09100-z | Y | Y | Y | Y | Y | 5 |
| (Aujnarain et al., 2016) Paradoxical hyperhidrosis in a patient with ectodermal dysplasia and immunodeficiency | 10.14785/lymphosign-2016-0002 | Y | Y | Y | N | Y | 4 |
| (Bayart et al., 2018) Pilocytic astrocytoma with leptomeningeal spread in a patient with incontinentia pigmenti presenting with unilateral nystagmus | 10.1002/pbc.26886 | N | Y | Y | N | Y | 3 |
| (Bryant & Rutledge, 2007) Abnormal white matter in a neurologically intact child with incontinentia pigmenti | 10.1016/j.pediatrneurol.2006.11.009 | Y | Y | Y | Y | Y | 5 |
| (Callea et al., 2011) Dental phenotype in a patient with hypoidrotic ectodermal dysplasia and severe immunodeficiency | https://dergipark.org.tr/en/download/article-file/91370 | Y | Y | Y | N | Y | 4 |
| (Carlberg et al., 2014) Hypohidrotic ectodermal dysplasia, osteopetrosis, lymphedema, and immunodeficiency in an infant with multiple opportunistic infections | 10.1111/pde.12103 | Y | Y | Y | N | Y | 4 |
| (Chang et al., 2008) A male infant with anhidrotic ectodermal dysplasia/immunodeficiency accompanied by incontinentia pigmenti and a mutation in the NEMO pathway | 10.1016/j.jaad.2007.02.024 | N | Y | N | N | Y | 2 |
| (Čulić et al., 2008) De novo NEMO gene deletion (D4-10) - A cause of incontinentia pigmenti in a female infant: A case report | https://hrcak.srce.hr/file/54600 | Y | Y | N | N | Y | 3 |
| (Danescu et al., 2018) A novel IKBKG mutation in a patient with incontinentia pigmenti and features of hepatic ciliopathy | 10.1111/ajd.12805 | Y | Y | N | N | Y | 3 |
| (de Jesus et al., 2020) Distinct interferon signatures and cytokine patterns define additional systemic autoinflammatory diseases | 10.1172/JCI129301 | N | Y | N | N | Y | 2 |
| (Devora et al., 2010) A Novel Missense Mutation in the Nuclear Factor-kappa B Essential Modulator (NEMO) Gene Resulting in Impaired Activation of the NF-kappa B Pathway and a Unique Clinical Phenotype Presenting as MRSA Subdural Empyema | 10.1007/s10875-010-9445-y | Y | Y | N | N | Y | 3 |
| (Dufke et al., 2001) Hydrops fetalis in three male fetuses of a female with incontinentia pigmenti | 10.1002/pd.165 | Y | Y | N | N | Y | 3 |
| (Dupuis-Girod et al., 2002) Osteopetrosis, lymphedema, anhidrotic ectodermal dysplasia, and immunodeficiency in a boy and incontinentia pigmenti in his mother | 10.1542/peds.109.6.e97 | Y | Y | Y | Y | Y | 5 |
| (Ergin et al., 2022) A Turkish case of incontinentia pigmenti with a deletion mutation at Inhibitor of kappa B kinase gamma gene | 10.1186/s43042-022-00215-x | Y | Y | Y | N | Y | 4 |
| (Franco et al., 2006) Incontinentia pigmenti in a boy with XXY mosaicism detected by fluorescence in situ hybridization | 10.1016/j.jaad.2005.11.1068 | Y | N | Y | N | Y | 3 |
| (Fusco et al., 2017) Unusual father-to-daughter transmission of incontinentia pigmenti due to mosaicism in IP males | 10.1542/peds.2016-2950 | Y | Y | Y | N | Y | 4 |
| (Gregersen et al., 2013) Diagnostic and molecular genetic challenges in male incontinentia pigmenti: A case report | 10.2340/00015555-1593 | Y | Y | Y | Y | Y | 5 |
| (Guevara et al., 2016) Improved molecular diagnosis of the common recurrent intragenic deletion mutation in IKBKG in a Filipino family with incontinentia pigmenti | 10.1111/ajd.12407 | Y | Y | Y | N | Y | 4 |
| (Haque et al., 2021) Analysis of IKBKG/NEMO gene in five Japanese cases of incontinentia pigmenti with retinopathy: fine genomic assay of a rare male case with mosaicism | 10.1038/s10038-020-00836-3 | Y | Y | Y | N | Y | 4 |
| (Heller et al., 2020) T Cell Impairment Is Predictive for a Severe Clinical Course in NEMO Deficiency | 10.1007/s10875-019-00728-y | Y | Y | Y | Y | Y | 5 |
| (Hegazy et al., 2022) NEMO-NDAS: A Panniculitis in the Young Representing an Autoinflammatory Disorder in Disguise | 10.1097/DAD.0000000000002144 | Y | Y | Y | N | Y | 4 |
| (Hsu et al., 2018) IKBKG (NEMO) 5' Untranslated Splice Mutations Lead to Severe, Chronic Disseminated Mycobacterial Infections | 10.1093/cid/ciy186 | Y | Y | Y | Y | Y | 5 |
| (Huang et al., 2007) A case of incontinentia pigmenti in Japan and its genetic examination | 10.1007/s10384-006-0412-3 | Y | Y | Y | N | Y | 4 |
| (Huang et al., 2015) Incontinentia pigmenti associated with seizures: A case report and literature review | https://www.researchgate.net/profile/Chih-Fen-Hu/publication/292551681_Incontinentia_Pigmenti_Associated_with_Seizures_A_Case_Report_and_Literature_Review/links/57c6e6b108aec24de042a242/Incontinentia-Pigmenti-Associated-with-Seizures-A-Case-Report-and-Literature-Review.pdf | Y | Y | Y | Y | Y | 5 |
| (Hubeau et al., 2011) New mechanism of X-linked anhidrotic ectodermal dysplasia with immunodeficiency: impairment of ubiquitin binding despite normal folding of NEMO protein | 10.1182/blood-2010-10-315234 | Y | Y | Y | Y | Y | 5 |
| (Hull et al., 2015) Somatic mosaicism of a novel IKBKG mutation in a male patient with incontinentia pigmenti | 10.1002/ajmg.a.37004 | Y | Y | Y | N | Y | 4 |
| (Huttner et al., 2010) Incontinetia pigmenti-related myopathy or unsolved "double trouble"? | 10.1016/j.nmd.2009.12.006 | Y | Y | Y | N | Y | 4 |
| (Imamura et al., 2011) Disseminated BCG infection mimicking metastatic nasopharyngeal carcinoma in an immunodeficient child with a novel hypomorphic NEMO mutation | 10.1007/s10875-011-9568-9 | Y | Y | Y | Y | Y | 5 |
| (Jain et al., 2001) Specific missense mutations in NEMO result in hyper-IgM syndrome with hypohydrotic ectodermal dysplasia | 10.1038/85277 | Y | Y | Y | N | Y | 4 |
| (Inaba et al., 2021) Case Report: Analysis of Preserved Umbilical Cord Clarified X-Linked Anhidrotic Ectodermal Dysplasia With Immunodeficiency in Deceased, Undiagnosed Uncles | 10.3389/fimmu.2021.786164 | Y | Y | Y | N | Y | 4 |
| (Johnston et al., 2016) A Novel Mutation in IKBKG/NEMO Leads to Ectodermal Dysplasia with Severe Immunodeficiency (EDA-ID) | 10.1007/s10875-016-0309-y | Y | Y | Y | N | Y | 4 |
| (Kanai et al., 2021) Late-onset cerebral arteriopathy in a patient with incontinentia pigmenti | 10.1016/j.braindev.2020.12.015 | Y | Y | Y | Y | Y | 5 |
| (Karakawa et al., 2011) Decreased Expression in Nuclear Factor-kappa B Essential Modulator Due to a Novel Splice-Site Mutation Causes X-linked Ectodermal Dysplasia with Immunodeficiency | 10.1007/s10875-011-9560-4 | Y | Y | Y | N | Y | 4 |
| (Karamchandani-Patel et al., 2011) Congenital alterations of NEMO glutamic acid 223 result in hypohidrotic ectodermal dysplasia and immunodeficiency with normal serum IgG levels | 10.1016/j.anai.2011.03.009 | Y | Y | Y | N | Y | 4 |
| (Kawai et al., 2022) Incontinentia pigmenti inherited from a father with a low level atypical IKBKG deletion mosaicism: a case report | 10.1186/s12887-022-03444-6 | Y | Y | Y | N | Y | 4 |
| (Keller et al., 2011) Hypohidrotic ectodermal dysplasia and immunodeficiency with coincident NEMO and EDA mutations | 10.3389/fimmu.2011.00061 | Y | Y | Y | Y | Y | 5 |
| (Khan et al., 2016) Interferon-gamma reduces the proliferation of M-tuberculosis within macrophages from a patient with a novel hypomorphic NEMO mutation | 10.1002/pbc.26098 | Y | Y | Y | N | Y | 4 |
| (Kim et al., 2014) A healthy delivery of twins by assisted reproduction followed by preimplantation genetic screening in a woman with X-linked dominant incontinentia pigmenti | 10.5653/cerm.2014.41.4.168 | Y | Y | Y | N | Y | 4 |
| (Kibbi et al., 2018) A case of subungual tumors of incontinentia pigmenti: A rare manifestation and association with bipolar disease | 10.1016/j.jdcr.2018.03.018 | Y | Y | Y | N | Y | 4 |
| (Kiritsi et al., 2016) The mysteries of mosaicism: Phenotypic variability in a family with incontinentia pigmenti | 10.1684/ejd.2016.2829 | Y | Y | Y | N | Y | 4 |
| (Klemann et al., 2016) Transplantation from a symptomatic carrier sister restores host defenses but does not prevent colitis in NEMO deficiency | 10.1016/j.clim.2016.01.010 | Y | Y | Y | Y | Y | 5 |
| (Kmetz et al., 2009) Incontinentia pigmenti with a foreshortened hand: Evidence for the significance of NFκB in human morphogenesis | 10.1111/j.1525-1470.2008.00829.x | Y | Y | Y | N | Y | 4 |
| (Kolitz et al., 2021) A novel NEMO/IKBKG mutation identified in a primary immunodeficiency disorder with recurrent atypical mycobacterial infections | 10.1016/j.jdcr.2020.10.019 | Y | Y | Y | N | Y | 4 |
| (Ku et al., 2005) NEMO mutations in 2 unrelated boys with severe infections and conical teeth | 10.1542/peds.2004-1754 | Y | Y | Y | N | Y | 4 |
| (Ku et al., 2007) IRAK4 and NEMO mutations in otherwise healthy children with recurrent invasive pneumococcal disease | 10.1136/jmg.2006.044446 | Y | Y | Y | Y | Y | 5 |
| (Lee et al., 2011) Incontinentia pigmenti in a newborn with NEMO mutation | 10.3346/jkms.2011.26.2.308 | Y | Y | Y | N | Y | 4 |
| (Loh et al., 2008) A genetic cause for neonatal encephalopathy: incontinentia pigmenti with NEMO mutation | 10.1111/j.1651-2227.2007.00630.x | Y | Y | Y | N | Y | 4 |
| (Maingay-de Groof et al., 2008) Extensive cerebral infarction in the newborn due to incontinentia pigmenti | 10.1016/j.ejpn.2007.09.001 | Y | Y | Y | N | Y | 4 |
| (Mancini et al., 2008) X-linked ectodermal dysplasia with immunodeficiency caused by NEMO mutation: early recognition and diagnosis | 10.1001/archderm.144.3.342 | Y | Y | Y | N | Y | 4 |
| (Mansour et al., 2001) Rapid publication - Incontinentia pigmenti in a surviving male is accompanied by hypohidrotic ectodermal dysplasia and recurrent infection | 10.1002/1096-8628(2001)9999:9999 | Y | Y | Y | Y | Y | 5 |
| (Margari et al., 2013) Long-term follow-up of neurological manifestations in a boy with incontinentia pigmenti | 10.1007/s00431-013-2021-8 | Y | Y | Y | Y | Y | 5 |
| (Martinez-Pomar et al., 2005) A new mutation in exon 7 of NEMO gene: Late skewed X-chromosome inactivation in an incontinentia pigmenti female patient with immunodeficiency | 10.1007/s00439-005-0068-y | Y | Y | Y | Y | Y | 5 |
| (Martuszewski et al., 2020) Successful Allogeneic Stem Cell Transplantation in Nuclear Factor-Kappa B Essential Modulator Deficiency Syndrome After Treosulfan-Based Conditioning: A Case Report | 10.1016/j.transproceed.2019.11.033 | Y | Y | Y | N | Y | 4 |
| (Matsumoto et al., 2009) Acute disseminated encephalomyelitis in an infant with incontinentia pigmenti | 10.1016/j.braindev.2008.08.010 | Y | Y | Y | N | Y | 4 |
| (Matsuzaki et al., 2018) Incontinentia pigmenti in a male (XY) infant with long-term follow up over 8 years | 10.1111/1346-8138.14002 | N | Y | Y | Y | Y | 4 |
| (Mayer et al., 2003) Novel corneal features in two males with incontinentia pigmenti | 10.1136/bjo.87.5.554 | Y | N | Y | N | Y | 3 |
| (Minakawa et al., 2009) Successful umbilical cord blood transplantation for intractable eczematous eruption in hypohidrotic ectodermal dysplasia with immunodeficiency | 10.1111/j.1365-2230.2009.03473.x | Y | Y | Y | N | Y | 4 |
| (Minić et al., 2013) First IKBKG gene mutation study in Serbian incontinentia pigmenti patients | 10.2298/sarh1308490m | Y | Y | Y | N | Y | 4 |
| (Minić et al., 2022) Challenges in Rare Diseases Diagnostics: Incontinentia Pigmenti with Heterozygous GBA Mutation | 10.3390/diagnostics12071711 | N | Y | Y | N | Y | 3 |
| (Minić et al., 2015) A Novel Frameshift Mutation of the IKBKG Gene Causing Typical Incontinentia Pigmenti | 10.2298/sarh1512752m | Y | Y | Y | Y | Y | 5 |
| (Mizukami et al., 2012) Successful treatment with infliximab for inflammatory colitis in a patient with X-linked anhidrotic ectodermal dysplasia with immunodeficiency | 10.1007/s10875-011-9600-0 | Y | Y | Y | Y | Y | 5 |
| (Mizuno et al., 2020) A successful treatment of tadalafil in incontinentia pigmenti with pulmonary hypertension | 10.1016/j.ejmg.2019.103764 | N | Y | Y | N | Y | 3 |
| (Moosajee et al., 2018) Retinal Angiography Findings in Male Infant With Incontinentia Pigmenti and Sickle Cell Trait | 10.1001/jamaophthalmol.2018.3140 | Y | Y | Y | N | N | 3 |
| (Moro et al., 2020) Incontinentia Pigmenti Associated with Aplasia Cutis Congenita in a Newborn Male with Klinefelter Syndrome: Is the Severity of Neurological Involvement Linked to Skin Manifestations? | 10.1007/s13555-019-00336-z | Y | Y | Y | N | Y | 4 |
| (Mullan et al., 2014) Incontinentia pigmenti in an XY Boy: Case report and review of the literature | 10.2310/7750.2013.13036 | Y | Y | Y | N | Y | 4 |
| (Nicolaou & Graham‐Brown, 2003) Nail dystrophy, an unusual presentation of incontinentia pigmenti | 10.1111/j.1365-2133.2003.05694.x | Y | Y | Y | N | Y | 4 |
| (Nishikomori et al., 2004) X-linked ectodermal dysplasia and immunodeficiency caused by reversion mosaicism of NEMO reveals a critical role for NEMO in human T-cell development and/or survival | 10.1182/blood-2003-10-3655 | Y | Y | Y | N | Y | 4 |
| (Ogasawara et al., 2019) Corticosteroid Therapy in Neonatal Incontinentia Pigmenti With Asymptomatic Cerebral Lesions | 10.1016/j.pediatrneurol.2019.04.003 | Y | Y | Y | N | Y | 4 |
| (Ohnishi et al., 2017) Immunodeficiency in Two Female Patients with Incontinentia Pigmenti with Heterozygous NEMO Mutation Diagnosed by LPS Unresponsiveness | 10.1007/s10875-017-0417-3 | Y | Y | Y | Y | Y | 5 |
| (Onnis et al., 2018) Cardiopulmonary anomalies in incontinentia pigmenti patients | 10.1111/ijd.13835 | Y | Y | Y | N | Y | 4 |
| (Orange et al., 2002) Deficient natural killer cell cytotoxicity in patients with IKK-gamma/NEMO mutations | 10.1172/JCI14858 | N | Y | Y | Y | Y | 4 |
| (Orange, Levy, et al., 2004) Human nuclear factor κb essential modulator mutation can result in immunodeficiency without ectodermal dysplasia | 10.1016/j.jaci.2004.06.052 | Y | Y | Y | N | Y | 4 |
| (Ørstavik et al., 2006) Novel splicing mutation in the NEMO (IKK-gamma) gene with severe immunodeficiency and heterogeneity of X-chromosome inactivation | 10.1002/ajmg.a.31026 | Y | Y | Y | Y | Y | 5 |
| (Pai et al., 2008) Allogeneic transplantation successfully corrects immune defects, but not susceptibility to colitis, in a patient with nuclear factor-kappaB essential modulator deficiency | 10.1016/j.jaci.2008.08.026 | Y | Y | Y | Y | Y | 5 |
| (Pauly et al., 2005) Incontinentia pigmenti in combination with decreased IgG subclass concentrations in a female newborn | 10.1159/000087416 | Y | Y | Y | Y | Y | 5 |
| (Pengelly et al., 2015) Resolving clinical diagnoses for syndromic cleft lip and/or palate phenotypes using whole-exome sequencing | 10.1111/cge.12547 | Y | Y | Y | N | Y | 4 |
| (Permaul et al., 2009) Allogeneic hematopoietic stem cell transplantation for X-linked ectodermal dysplasia and immunodeficiency: Case report and review of outcomes | 10.1007/s12026-008-8085-2 | Y | Y | Y | Y | Y | 5 |
| (Piccoli et al., 2012) NEMO syndrome (incontinentia pigmenti) and systemic lupus erythematosus: A new disease association | 10.1177/0961203311433140 | Y | Y | Y | N | Y | 4 |
| (Ramírez-Alejo et al., 2015) Novel hypomorphic mutation in IKBKG impairs NEMO-ubiquitylation causing ectodermal dysplasia, immunodeficiency, incontinentia pigmenti, and immune thrombocytopenic purpura | 10.1016/j.clim.2015.06.007 | Y | Y | Y | N | Y | 4 |
| (Rashidghamat et al., 2016) Incontinentia pigmenti in a father and daughter | 10.1111/bjd.14615 | Y | Y | Y | N | Y | 4 |
| (Rheault, 2021) Severe COVID-19 and long COVID in a 31-year-old woman with incontinentia pigmenti: A case report | 10.1177/2050313X211059295 | Y | Y | Y | N | Y | 4 |
| (Roberts et al., 2010) A novel NEMO gene mutation causing osteopetrosis, lymphoedema, hypohidrotic ectodermal dysplasia and immunodeficiency (OL-HED-ID) | 10.1007/s00431-010-1206-7 | Y | Y | Y | Y | Y | 5 |
| (Salt et al., 2008) IKBKG (nuclear factor-kappa B essential modulator) mutation can be associated with opportunistic infection without impairing Toll-like receptor function | 10.1016/j.jaci.2007.11.014 | Y | Y | Y | N | Y | 4 |
| (Sanka & Kumar, 2004) An unusual newborn rash | 10.1080/15227950490923750 | Y | Y | Y | N | Y | 4 |
| (Schmid et al., 2006) Transient hemophagocytosis with deficient cellular cytotoxicity, monoclonal immunoglobulin M gammopathy, increased T-cell numbers, and hypomorphic NEMO mutation | 10.1542/peds.2005-2062 | Y | Y | Y | Y | Y | 5 |
| (Seo et al., 2017) A 6-Month-Old Girl with Incontinentia Pigmenti Presenting as Status Epilepticus | 10.14581/jer.17019 | Y | Y | Y | N | Y | 4 |
| (Silan et al., 2004) Incontinentia pigmenti with NEMO mutation in a Turkish family | 10.1111/j.1365-4632.2004.02156.x | Y | Y | Y | N | Y | 4 |
| (Song et al., 2010) The common NF-κB essential modulator (NEMO) gene rearrangement in Korean patients with incontinentia pigmenti | 10.3346/jkms.2010.25.10.1513 | Y | Y | Y | N | Y | 4 |
| (Sun et al., 2019) A novel inhibitor of nuclear factor kappa-B kinase subunit gamma mutation identified in an incontinentia pigmenti patient with syndromic tooth agenesis | 10.1016/j.archoralbio.2019.03.013 | Y | Y | Y | N | Y | 4 |
| (Takada et al., 2010) NEMO mutation as a cause of familial occurrence of Beh?et's disease in female patients | 10.1111/j.1399-0004.2010.01432.x | Y | Y | Y | N | Y | 4 |
| (Tono et al., 2007) Correction of immunodeficiency associated with NEMO mutation by umbilical cord blood transplantation using a reduced-intensity conditioning regimen | 10.1038/sj.bmt.1705658 | Y | Y | Y | Y | Y | 5 |
| (Toyohara et al., 2021) An infant with X-linked anhidrotic ectodermal dysplasia with immunodeficiency presenting with Pneumocystis pneumonia: A case report | 10.1002/ccr3.5093 | Y | Y | Y | N | Y | 4 |
| (Türkmen et al., 2007) A rare cause of neonatal seizure: Incontinentia pigmenti | PMID: 17990592 | Y | Y | Y | N | Y | 4 |
| (Veronese et al., 2018) A female newborn with papulovesicular lesions | 10.1111/ddg.13643 | Y | Y | Y | N | Y | 4 |
| (Wang et al., 2013) A 14-year-old girl with an unusual combination of incontinentia pigmenti and conversion disorder | PMID: 24260612 | N | Y | Y | N | Y | 3 |
| (Wolf et al., 2015) High-dose glucocorticoid therapy in the management of seizures in neonatal incontinentia pigmenti: a case report | 10.1177/0883073813517509 | Y | Y | Y | Y | Y | 5 |
| (Surucu Yilmaz et al., 2022) Low Density Granulocytes and Dysregulated Neutrophils Driving Autoinflammatory Manifestations in NEMO Deficiency | 10.1007/s10875-021-01176-3 | Y | Y | Y | Y | Y | 5 |
| (Zafeiriou et al., 2013) Incontinentia pigmenti: A skin, brain, and eye matter | 10.1016/j.jpeds.2013.06.029 | Y | N | Y | N | Y | 3 |
| (Zhang et al., 2014) Unusual hyperpigmented patches, an undeveloped breast and a cataract in a female with incontinentia pigmenti | 10.1111/1346-8138.12285 | Y | Y | Y | N | Y | 4 |
| (Su et al., 2004) De novo incontinentia pigmenti in female twins | PMID: 15493740 | Y | Y | Y | N | Y | 4 |
| (Jiang et al., 2022) NEMO Gene Mutations in Two Chinese Females with Incontinentia Pigmenti | 10.2147/CCID.S363683 | Y | Y | Y | N | Y | 4 |
| (Williams et al., 2017) Incontinentia pigmenti, an x-linked dominant disorder, in a 2-year-old boy with Klinefelter syndrome | 10.4103/IJPM.IJPM_91_16 | Y | Y | Y | N | Y | 4 |
| (Azarbayjani et al., 2021) A report of incontinentia pigmenti in an 11-year-old girl | 10.5812/ijp.103348 | Y | Y | Y | Y | Y | 5 |
| (Mansour et al., 2001) Incontinentia pigmenti in a surviving male is accompanied by hypohidrotic ectodermal dysplasia and recurrent infection | 10.1002/1096-8628(2001)9999:9999<::aid-ajmg1155>3.0.co;2-y | Y | Y | Y | Y | Y | 5 |
| (Pizzamiglio et al., 2014) Incontinentia Pigmenti: Learning disabilities are a fundamental hallmark of the disease | 10.1371/journal.pone.0087771 | N | Y | N | N | Y | 2 |
| (Niehues et al., 2004) Nuclear factor kappaB essential modulator-deficient child with immunodeficiency yet without anhidrotic ectodermal dysplasia | 10.1016/j.jaci.2004.08.047 | Y | Y | Y | Y | Y | 5 |
| (Ricci et al., 2017) OL-EDA-ID Syndrome: a Novel Hypomorphic NEMO Mutation Associated with a Severe Clinical Presentation and Transient HLH | 10.1007/s10875-016-0350-x | Y | Y | Y | Y | Y | 5 |
| (Filipe-Santos et al., 2006) X-linked susceptibility to mycobacteria is caused by mutations in NEMO impairing CD40-dependent IL-12 production | 10.1084/jem.20060085 | Y | Y | Y | Y | Y | 5 |
| (Azarsiz et al., 2023) Eight years of follow-up experience in children with mendelian susceptibility to mycobacterial disease and review of the literature | 10.12932/AP-271219-0726 | Y | Y | N | Y | Y | 4 |
| (Inoue et al., 2018) Cutaneous squamous cell carcinoma, thyroid cancer and Langerhans cell histiocytosis in a patient with X-linked recessive Mendelian susceptibility to mycobacterial diseases with a nuclear factor-κB essential modifier mutation | 10.1111/1346-8138.14482 | Y | Y | Y | Y | Y | 5 |
| (Rae et al., 2017) Autoimmunity/inflammation in a monogenic primary immunodeficiency cohort | 10.1038/cti.2017.38 | Y | Y | N | N | Y | 3 |

**Supplementary Table 1B.** Quality and risk assessment of case control studies.

| **(Author and Date) Studies** | **DOI** | **Selection** | **Comparability** | **Exposure** | **Total** |
| --- | --- | --- | --- | --- | --- |
| (Frost et al., 2019) Absence of an osteopetrosis phenotype in IKBKG (NEMO) mutation-positive women: A case-control study | 10.1016/j.bone.2019.01.014 | 4 | 1 | 3 | 8 |
| (Lee et al., 2022) Genetically programmed alternative splicing of NEMO mediates an autoinflammatory disease phenotype | 10.1172/JCI128808 | 3 | 1 | 0 | 4 |

Selection contains: 1. Is the case definition adequate; 2. Representativeness of the cases; 3. Selection of Controls ; 4. Definition of Controls; Comparability means: Comparability of cases and controls on the basis of the design or analysis; Outcome contains: 1. Ascertainment of exposure; 2. Same method of ascertainment for cases and controls; 3. Non-Response rate. One point is awarded for meeting any of the above assessments.

**Supplementary Table 1C.** Quality and risk assessment of cohort studies.

| **(Author and Date) Studies** | **DOI** | **Selection** | **Comparability** | **Outcome** | **Total** |
| --- | --- | --- | --- | --- | --- |
| (Fusco et al., 2004) Molecular analysis of the genetic defect in a large cohort of IP patients and identification of novel NEMO mutations interfering with NF-κB activation | 10.1093/hmg/ddh192 | 3 | 1 | 2 | 5 |
| (Fusco et al., 2007) Clinical diagnosis of incontinentia pigmenti in a cohort of male patients | 10.1016/j.jaad.2006.09.019 | 2 | 1 | 1 | 4 |

Selection contains: 1. Representativeness of the exposed; 2. Selection of the non exposed cohort; 3. Ascertainment of exposure; 4.Demonstration that outcome of interest was not present at start of study; Comparability means: Comparability of cohorts on the basis of the design or analysis; Outcome contains: 1. Assessment of outcome; 2. Was follow-up long enough for outcomes to occur; 3. Adequacy of follow up of cohorts. One point is awarded for meeting any of the above assessments
