# Supplementary material for: Clinical relevance of loss-of-function mutations of NEMO/IKBKG
Source: Genes Dis. 2025 Jan 12;12(5):101531. doi: 10.1016/j.gendis.2025.101531 (PMC12221755; doi:10.1016/j.gendis.2025.101531)
Supplement: Multimedia component 3 [file mmc3.docx]

**Supplementary table 2.** Detailed classification list of clinical findings for patients with IP and EDA-ID

|  |  | **IP** | **EDA-ID** |
| --- | --- | --- | --- |
| **Eye** | Tracitional retinal detachment | 34 | 1 |
|  | Vision defects | 29 | 1 |
|  | Haemorrhage | 18 | 1 |
|  | Strabismus | 15 | 2 |
|  | Non-perfusion | 14 | 0 |
|  | Neovascularization | 8 | 0 |
|  | Vascular tortuosity | 6 | 0 |
|  | Pigment anomaly | 6 | 0 |
|  | Cataracts | 6 | 0 |
|  | Optic nerves anomalies | 3 | 0 |
|  | 0thers | 3 nystagmus;  3 malformation;  2 periorbital wrinkling;  1 glaucoma;  1 iriscyst;  1 arteriovenous;  1 macular alteration; | 1 chronic uveitis;  1 pupil deformation |
| **Hair** | Alopecia | 99 | 3 |
|  | Thin hair | 37 | 43 |
|  | Woolly hair | 17 | 2 |
|  | Hypertrichosis | 2 | 0 |
| **Teeth** | Shape anomailies | 56 | 47 |
|  | Agenesis | 47 | 22 |
|  | Delayed dentition | 31 | 5 |
|  | Dental dystrophy | 5 | 12 |
|  | Cariotic teeth | 2 | 2 |
|  | Others | 6 chronic gingivitis;  4 ectopic tooth in the  hard palate;  3 mandibular deformity;  2 extra teeth;  2 focal gingival hypoplasia;  1 fused teeth | 0 |
| **Prefrons** | Frontal bossing | 0 | 5 |
| **Skin** | Pigmentation | 394 | 11 |
|  | Eczma | 0 | 6 |
|  | Hyperkeratosis | 0 | 3 |
|  | Dry | 0 | 10 |
|  | Rash or erythematous or pustules | 0 | 10 |
|  | hypohidrosis/anhidrosis | 0 | 24 |
|  | Others | 1 Thin translucent skin;  1 Abcsess | 0 |
| **Breast** | Nipple abnormal | 9 | 0 |
|  | Nipple abnormal | 3 | 0 |
| **Nose** | Depress nasal bridge | 0 | 2 |
|  | Saddle nose | 0 | 1 |
| **CNS** | Seizures | 63 | 4 |
|  | Motor impairment | 24 | 0 |
|  | Intelligence disability | 17 | 1 |
|  | Infarcts | 2 | 2 |
|  | Others | 2 hydrocephaly;  2 learning difficult;  2 Congenital malfomation;  2 space occupying lesion;  1 Periventricular;  1 brain atrophy;  1 striatal arteriopathy;  1 Arachnoid cysts;  1 Partial agenesis of  the corpus callosum cerebellar ataxia  1 leukomalacia;  1 Arachnoid cysts;  1 subarachnoid haemorrhage; | 0 |
| **Nail** | Dystrophy | 0 | 27 |
|  | Punctuate  depressions | 0 | 3 |
|  | Onychogryphosis | 0 | 2 |
|  | Nail clubbing | 0 | 1 |
| **Palate** | High-arched palate | 7 | 1 |
|  | Cleft of palate | 0 | 1 |
