# Supplementary material for: Clinical relevance of loss-of-function mutations of NEMO/IKBKG
Source: Genes Dis. 2025 Jan 12;12(5):101531. doi: 10.1016/j.gendis.2025.101531 (PMC12221755; doi:10.1016/j.gendis.2025.101531)
Supplement: Multimedia component 4 [file mmc4.docx]

**Supplementary table 3.** Abnormal immune status of EDA-ID, ID and NDAS patients with NEMO mutation.

| Disease | Immune function | Abnormal level | n |
| --- | --- | --- | --- |
| EDA-ID | IgG | Low | 39 |
|  |  | High | 6 |
|  | IgM | Low | 21 |
|  |  | High | 11 |
|  | IgA | Low | 21 |
|  |  | High | 10 |
|  | B cell | Low | 3 |
|  |  | High | 5 |
|  | T cell | Low | 3 |
|  |  | High | 1 |
| ID | IgG | Low | 12 |
|  |  | High | 0 |
|  | IgM | C | 5 |
|  |  | High | 2 |
|  | IgA | Low | 5 |
|  |  | High | 1 |
|  | B cell | Low | 0 |
|  |  | High | 2 |
|  | T cell | Low | 2 |
|  |  | High | 0 |
| NDAS | IgG | Low | 6 |
|  |  | High | 0 |
|  | IgM | Low | 2 |
|  |  | High | 0 |
|  | IgA | Low | 1 |
|  |  | High | 0 |
|  | B cell | Low | 4 |
|  |  | High | 0 |
